# Sensory modality-specific wiring of thalamocortical circuits

**DOI:** 10.1038/s41583-025-00945-y

**Published:** 2025-07-30

**Authors:** Teresa Guillamón-Vivancos, Mar Aníbal-Martínez, Lorenzo Puche-Aroca, Francisco J Martini, Guillermina López-Bendito

**Affiliations:** Instituto de Neurociencias de Alicante, Universidad Miguel Hernández- Consejo Superior de Investigaciones Científicas (UMH-CSIC), San Juan de Alicante, Alicante, Spain

## Abstract

The thalamus is an essential element for sensory information processing, serving as a link between peripheral sensory stimuli and cortical circuits. Consequently, the development of thalamocortical projections has been a central focus in systems neuroscience. Although significant progress has been made in understanding the mechanisms guiding thalamic axon navigation from the diencephalon to the cortex, our understanding of the processes underlying sensory modality specificity in thalamocortical circuits remains incomplete. Modern genomic, physiological and imaging approaches have yielded exciting results, providing novel insights into the specialization of visual, somatosensory and auditory thalamocortical circuits. Recent findings have shed light on the genetic and spontaneous activity mechanisms involved in the formation of distinct sensory modalities, rekindling the interest in the thalamus and opening new research perspectives on the development of this diencephalic structure.

## Introduction

**Fig. 1:**
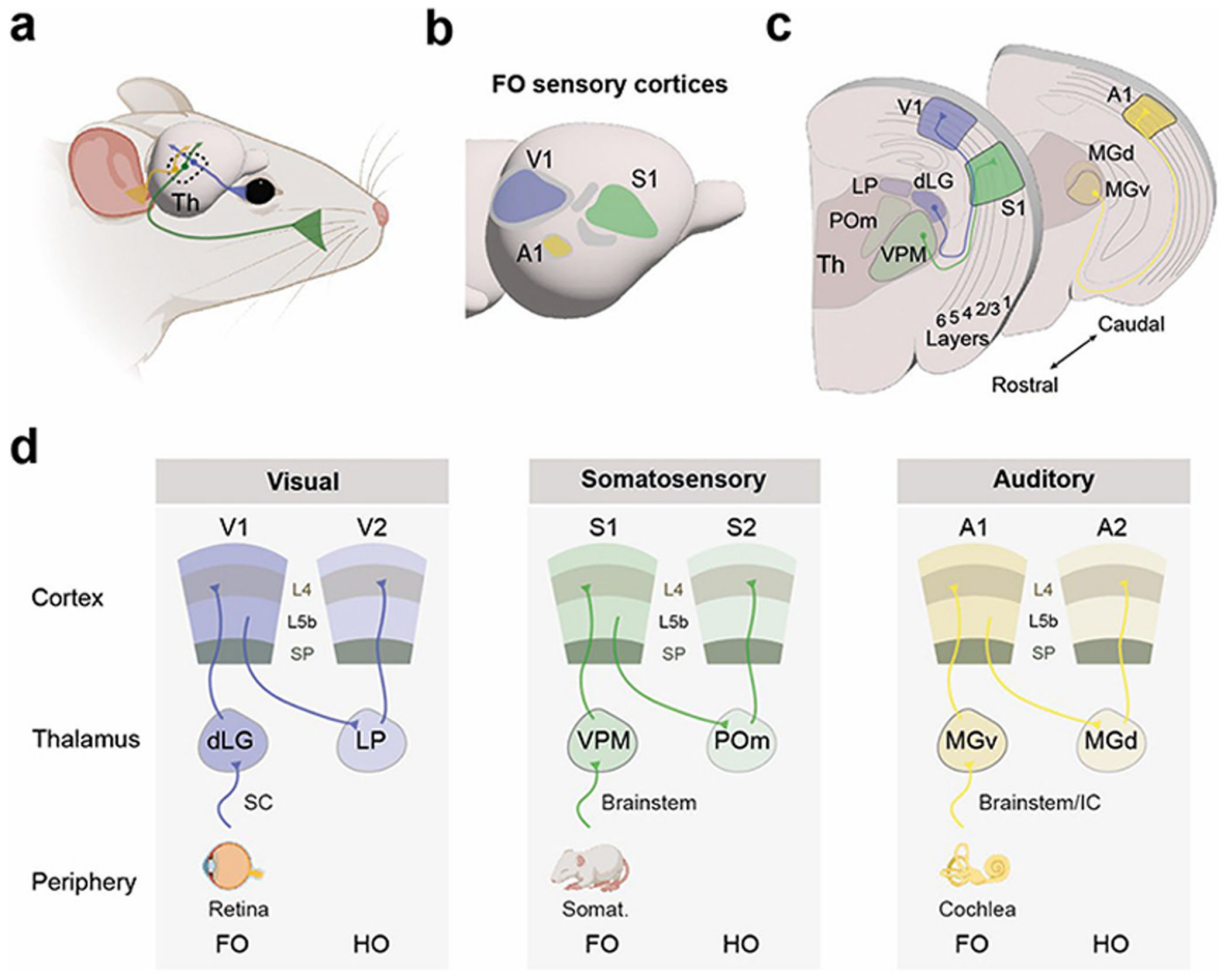
Distinct thalamic nuclei relay sensory-modality specific information to the corresponding sensory cortices. **a**, Input from the sensory organs (for example, whiskers, eye or ear) reaches the thalamus, which in turn relays information to the cortex. **b**, Lateral view of the mouse cortex highlighting the location of primary visual, somatosensory and auditory cortices. Secondary, higher-order areas are shown in gray. **c**, Coronal sections through the mouse brain showing the somatosensory projection from VPM to S1, in green, the visual projection from dLG to V1, in blue, and the auditory projection from MGv to A1. **d**, Schematic representation of three examples of thalamocortical loops: in each case (visual, somatosensory and auditory), input from the periphery reaches the first-order sensory nuclei of the thalamus, which project to primary sensory cortices. Primary sensory cortices project to higher-order nuclei, which in turn relay it to the secondary sensory areas. Abbreviations: A1, primary auditory cortex; A2, secondary auditory cortex; dLG, dorsal lateral geniculate nucleus; FO, first-order; HO, higher-order; IC, inferior colliculus; L4, layer 4; L5B, layer 5B; LP, lateral posterior nucleus; MGd, dorsal division of the medial geniculate body; MGv, ventral division of the medial geniculate body; POm, posterior medial nucleus; S1, primary somatosensory cortex; S2, secondary somatosensory cortex; SC, superior colliculus; V1, primary visual cortex; V2, secondary visual cortex; VPM, ventral posterior medial nucleus. Mouse, retina and cochlea images were created in BioRender. Martini, F. (2025) https://BioRender.com/m24d847.

The thalamus is a complex structure responsible for integrating information from diverse brain regions. Some of the major inputs to the thalamus originate from the cortex, basal ganglia, cerebellum, hypothalamus and superior colliculus, among others^1^. As a result, the thalamus serves as a key hub for various aspects of behavior, cognition, brain states, and sensory and motor functions^2^. These diverse inputs contribute to a mosaic-like organization in which the thalamus is subdivided into groups of cells forming nuclei. According to a classical functional classification, thalamic nuclei can be categorized into sensory, motor, association, and midline and intralaminar nuclei^3^. For decades, research has predominantly focused on the sensory thalamus, resulting in a vast body of knowledge regarding its anatomical and functional properties. The sensory thalamus receives, processes, and transmits information from the peripheral sensory organs to the corresponding regions of the sensory cortices^4,5^. Each thalamic nucleus is specialized for a particular sensory modality, defined by the type of sensory input it processes, such as visual, auditory, or somatosensory information. The so-called first-order nuclei (FO) directly process ascending information from a single sensory modality and relay it through their projection to layer 4 of the primary sensory cortices^6^. Higher-order nuclei (HO) handle more complex information, including inputs descending from sensory cortices and usually target secondary or associative sensory cortices^7,8^ (Box 1, Fig. 1).

The specificity of sensory modalities arises from both the connectivity patterns and the intrinsic properties of thalamic neurons^9–11^. Neurons within a given sensory nucleus share similar hodology as well as molecular and physiological properties^9^. These features are determined early in embryonic development, when thalamic neurons originate from a seemingly homogeneous progenitor pool^5,12^. This raises an important question: how do thalamic progenitor cells give rise to mature neurons with nucleus-specific properties and connectivity?

The emergence of sensory pathways in the thalamocortical system likely depends on a complex interplay of intrinsic and extrinsic factors. In this framework, intrinsic transcriptional programs within progenitor cells establish the foundation for neuronal specification, whereas spontaneous activity helps to shape and refine connectivity patterns. Although some aspects of the development of the thalamocortical system are well understood, the mechanisms underlying sensory specification remain less explored. Recent advances in high-throughput transcriptomics and activity recording techniques are shedding light on these processes, providing valuable insights into the factors that define sensory-specific connectivity.

This review explores the developmental mechanisms underlying the emergence of sensory pathways in the thalamocortical system, with an emphasis on the combination of genetic programs and activity-dependent processes that drive the specification of distinct sensory modalities.

## Development of thalamic sensory modality nuclei: Transcriptional control of neuronal identity

Traditionally, the identity of the distinct thalamic nuclei has been defined based on their anatomy and specific connectivity. However, recent studies have classified mature thalamic nuclei based on their transcriptional profiles^13,14^. Understanding how transcriptional landscape of thalamic nuclei emerges during development is likely to provide insights into the mechanisms of neural circuit formation. Below, the growing body of evidence will be summarized suggesting that these genetic patterns emerge early, well before sensory experiences, and are essential for the proper development and specification of thalamic circuits^15–18^.

### Early patterning and regionalization of the thalamus

**Fig. 2:**
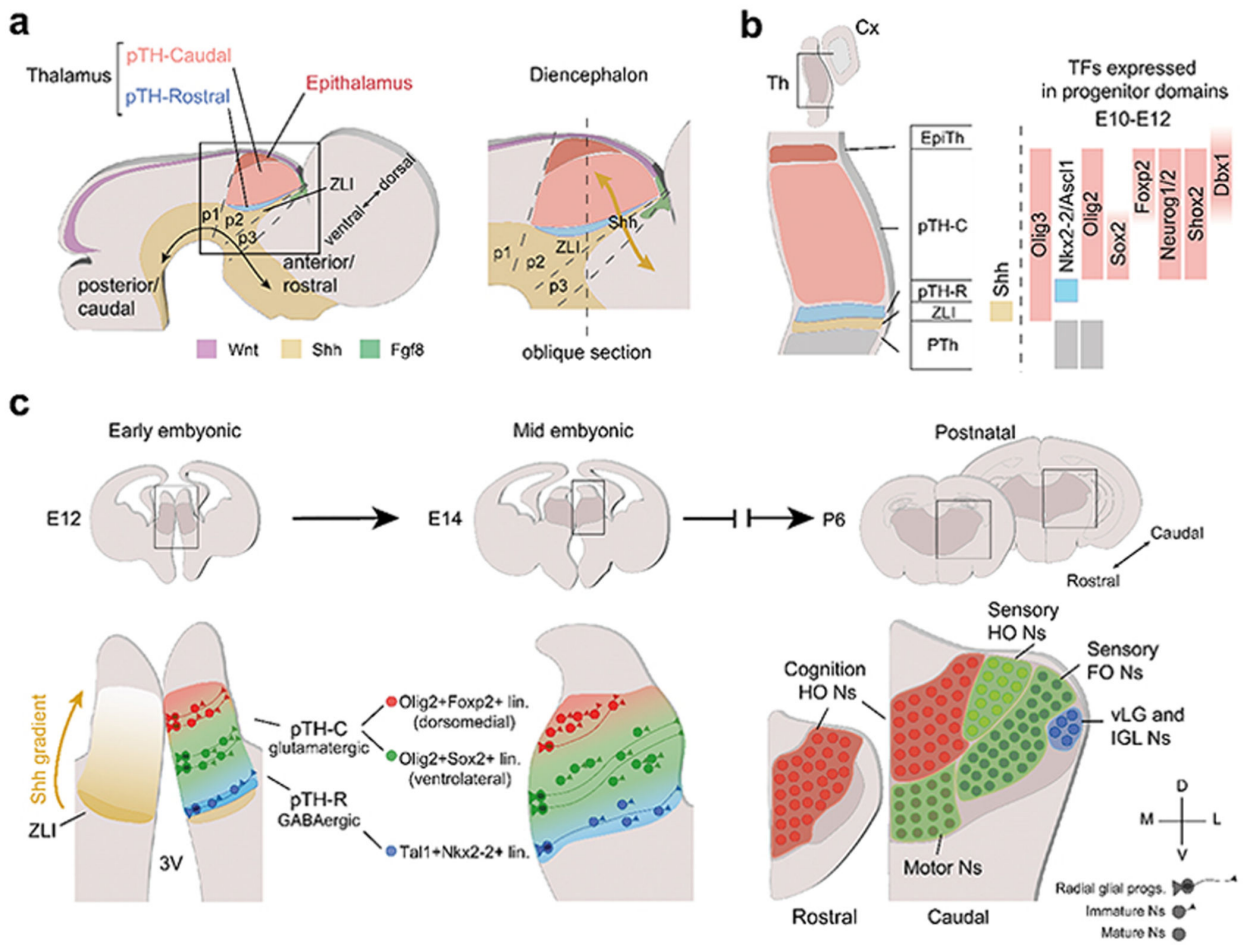
Thalamus formation and regionalization. **a**, Schema of sagittal section through the early developing mouse brain at E12 with prosomeres (p) 1-3 and key morphogens (Wnt, Shh and Fgf8) indicated. Inset: Black square indicates how the morphogen Shh expressed in the ZLI regulates the regionalization of the entire diencephalon. **b**, Oblique section of the diencephalon (from inset in **a**, vertical dashed line) showing different TFs expressed at distinct progenitor domains in early stages of thalamic development. **c**, Proposed model for thalamic nuclei specification from early radial glial progenitors to progressively mature neurons. Thalamic radial glial progenitors are organized rostro-caudally in different lineages and acquire distinct identities based on expression of several TFs. Different cell lineages contribute to different thalamic nuclei in a sequential temporal manner. Dots in right panel represent mature neurons. The schemas in the bottom row correspond to the squares depicted above, in coronal slices. Abbreviations: Cx, cortex; E, embryonic stage; EpiTh, epithalamus; FO, first-order; HO, higher-order; intergeniculate leaflet IGL; Ns, neurons; p, prosomeres; P, postnatal stage; PTh, prethalamus; pTH-C, caudal thalamic progenitor domain; pTH-R, rostral thalamic progenitor domain; TF, transcription factor; Th, thalamus; vLG, ventral lateral geniculate nucleus; ZLI, zona limitans intrathalamica; 3V, third ventricle.

The ontogenesis of the thalamus begins early in embryogenesis when the developing forebrain divides into anterior and posterior territories, known as the secondary prosencephalon and the diencephalon, respectively. Early patterning of the diencephalon is defined by sharp boundaries formed by key morphogens. According to the prosomeric model, morphogens divide the developing diencephalon into three transverse domains, or prosomeres (p), along the rostro-caudal axis of neural tube^19–23^. The rostral-most domain, p3, gives rise to the prethalamic nuclei (PTh), including the reticular thalamic nuclei, the ventral lateral geniculate nucleus (vLG) and zona incerta. The p2 domain, identified by the expression of the transcription factor Gbx2 in postmitotic neurons, generates the thalamus and the ephitalamus. Caudal to p2 appears p1, which gives rise to the pretectum. Separating the p2 and p3 regions is the zona limitans intrathalamica (ZLI), a distinct boundary marked by a core of *Shh*-expressing cells. Shh is a key morphogen that determines the transcriptional programs of recipient cells. Other morphogens or signalling molecules, like Wnt1, expressed dorsally and defining roof plate regions, and Fgf8, expressed rostrally, establish gradients that activate the regionalization of the diencephalon, including the thalamus^20,24^. This morphogen graded expression regulates the expression of region-specific transcription factors (TFs) that define the identity of the thalamic neurons (Fig. 2a). Some of these thalamic-specific TFs include Olig3, Ascl1, Nkx2-2, and Neurog1/2, which play critical roles in neuronal differentiation and regional specification. Specifically, *Olig3* marks thalamic progenitors in the ventricular zone^12,18,24,25^. The *Olig3*-expressing progenitor pool of the prospective thalamus is heterogeneous, comprising two subpopulations: the rostral (pTH-R) and the caudal (pTH-C) domains. In mice, the progenitors in pTH-R conform a small domain characterized by the expression of *Ascl1* (also called *Mash1*), *Nkx2-2*, and *Tal1*. These progenitors generate GABAergic projection neurons that populate intergeniculate leaflet (IGL) and the vLG. In contrast, pTH-C progenitors form a larger domain characterized by the expression of two basic helix-loop-helix TFs: Neurog1 and Neurog2. These progenitors generate glutamatergic cortical-projecting neurons that will populate the array of nuclei that form the mature thalamus^12,25,26^ (Fig. 2b).

### Transcriptional Programs of Thalamic Differentiation

Although significant progress has been made in understanding how specific progenitor cells divide and generate thalamocortical neurons that populate sensory modality-specific nuclei, numerous aspects of the molecular logic and developmental trajectories underlying the acquisition of the distinct sensory-modality identities remain to be fully elucidated.

### “Outside-in” model of thalamic neurogenesis

Recent use of clonal lineage tracing of thalamic progenitor cells has demonstrated that neurons generated at different timepoints contribute to distinct thalamic nuclei in a lateral-to-medial pattern. In particular, one of the first insights was achieved using MADM (Mosaic Analysis with Double Markers)^27^, a powerful lineage-tracing tool that enabled the tracking of single progenitors and their progeny to their final position, while also analyzing clonal dispersion of these cells. Firstly, Shi and colleagues^28^ identified subsets of neurons derived from individual thalamic progenitors that segregated ontogenetically based on their functional assignments to FO and HO nuclei. Building on this, Wong and colleagues^29^ combined the MADM approach with various CreER-expressing transgenic driver lines to demonstrate that neuronal clones are not only sorted by functionality, but also that spatial location of progenitor cells strongly correlates with the nuclei their progeny will populate. Notably, the FO and HO nuclei ontogeny unfolds in close proximity along the medial-lateral axis, suggesting they share a common lineage. In this framework, the authors found that the thalamic neurogenesis is characterized by a specific spatiotemporal pattern defined by a lateral-to-medial and ventral-to-dorsal gradient in which early-born neurons primarily contribute to the formation of primary sensory nuclei such as the ventral posterior (VP), dorsal LG (dLG), ventral medial geniculate (MGv), as well as latero-posterior (LP), and lateral dorsal (LD). These sensory thalamic nuclei encompass both FO and HO regions, reflecting a coordinated yet distinct process of nuclear specification. In contrast, late-born neurons occupy more dorso-medial thalamic nuclei such as mediodorsal (MD) and centromedial (CM) thalamic nuclei. This spatio-temporal organization has been referred to as the “outside-in” model of thalamic neurogenesis^30^.

#### Indirect neurogenesis in the thalamus

Furthermore, using Neurog1-CreER transgenic mice, which label intermediate progenitor cells (IPCs) of the pTH-C domain, Wong and colleagues showed that thalamic neurogenesis involves a prolonged and sequential specification of nuclear fates. In this model, each progenitor divides a limited number of times during a short neurogenic period, generating IPCs. These IPCs then divide once or twice to expand the neuronal population, supporting a sequential indirect neurogenic process and suggesting that indirect neurogenesis is vital to the development of thalamic nuclei. Supporting this, Guo and Li^31^ and Govek and colleagues^16^ used single-cell RNA sequencing (scRNA-seq) to identify distinct IPC populations in the diencephalon, revealing molecular heterogeneity within the progenitor pool. These findings align with earlier work by Wang and colleagues^32^, which first demonstrated the presence of IPCs in the thalamus and established their contribution to the glutamatergic neuron population. Indirect neurogenesis is a conserved mechanism, increasing in the cortex across evolution, especially in gyrencephalic mammals^33^. However, whether thalamic indirect neurogenesis scales with increasing brain complexity, as observed in the cortex, remains an open question. Together, these experiments show that thalamic progenitors could be partially pre-specified to generate FO or HO IPCs, which generate the neurons that progressively acquire their final fate, populating the diverse nuclei near their place of birth. Thus, thalamic nuclear identities seem to emerge through a tightly coordinated spatial and temporal model of specification, where the spatial arrangement of progenitors, combined with their sequential specification, guides the progressive acquisition of nuclear identity and final positional fate (Fig. 2c).

#### Shh-dependent and Shh-independent molecular trajectories

To further understand the molecular trajectories followed by progenitors during thalamic specification, Govek and colleagues^16^ provide a comprehensive scRNA-seq analysis of Shh-dependent and Shh-independent thalamic neuronal populations from embryonic stages (E) 12 to E18 of diencephalon development organized into distinct cell lineages corresponding to different groups of thalamic nuclei. Firstly, the authors identify 23 distinct cell populations, encompassing most known cell types in the caudal diencephalon. Focusing on progenitors, IPCs and neuronal population at four developmental stages (E12.5, E14.5, E16.5 and E18.5), they capture critical periods of thalamic proliferation, neurogenesis and differentiation. Their findings reveals that the pTH-R contains *Tal1*- *Nkx2-2*-expressing IPCs, which are Shh-dependent and generate the GABAergic neurons of the vLG and IGL. Conversely, glutamatergic neurons derive from the pTH-C. Specifically, the Shh-dependent the *Olig2*- and *Sox2*-expressing lineage generates the neurons that populate the ventrolateral regions: sensory nuclei, motor nuclei, and anterior nuclei related to cognition. The Shh-independent *Olig2*- and *Foxp2*-expressing IPCs give rise to neurons of the dorsomedial regions: the paraventricular nucleus and the nuclei projecting to the prefrontal cortex (MD and CM) (Fig. 2c). Supporting the “outside-in” neurogenesis model, Govek and colleagues show that ventrolateral nuclei are formed first, and dorsomedial nuclei are formed later. By mapping the distinct trajectories, the authors provide a detailed framework for how transcriptionally and spatially distinct progenitor populations generate the diverse neuronal types that establish thalamic nuclei. Their findings underscore the importance of Shh-dependent signaling and highlight the organizational complexity underpinning thalamic development.

#### First evidence of hierarchical order of sensory modalities

Furthermore, a transcriptional comparison of FO and HO nuclei of the somatosensory and visual modalities revealed that hierarchical order had a stronger impact on transcriptional identity of these nuclei than sensory modality^15^. This identity is, at least in part, determined by peripheral input, since peripheral input ablation induces HO-like transcriptional programs in FO nuclei, and causes abnormal projection from L5B neurons to FO thalamic neurons^15^. These results suggest that the HO configuration is the default feature, while FO-identity is dependent on peripheral input. Building upon this, research by Lo Giudice and colleagues^17^ using a high-temporal scRNAseq analysis spanning embryonic stages E11-E18, as well as postnatal stages P3 and P7, revealed both conserved and differential molecular programs governing the emergence of FO and HO neurons. Within these classes, specific molecular identities were identified for FO and HO visual and somatosensory nuclei. In this way, a key finding from this study is that, despite their concurrent birth, HO neurons begin differentiating earlier than FO neurons, highlighting a temporal distinction in their maturation programs. Furthermore, focusing specifically on the visual pathway, the authors demonstrated that the maturation of excitatory FO neurons relies on postnatal sensory input, whereas inhibitory neurons develop independently of such input. However, a previous study^34^ suggests that, while sensory input deprivation does not noticeably affect the transcriptional identity of the interneurons, visual input is critical for their migration and distribution within this nucleus as well as their synaptic integration into the visual thalamic circuits. These findings delineate distinct molecular pathways and input-dependent processes driving the differentiation of FO and HO neurons, confirming and extending previous results^15^.

#### Shared lineages between thalamic neurons and astrocytes

Transcriptional heterogeneity in the thalamus, however, may not be confined to neurons alone, but also extend to other cell types, such as astrocytes. Recent work by Herrero-Navarro and colleagues^35^ highlighted that both thalamic neurons and astrocytes share a common transcriptional identity rooted in their progenitors. Their study revealed that both cell types are specified at the progenitor level and emerge from the same progenitor groups, even within functionally distinct sensory nuclei. This shared genetic profile suggests that the differentiation of both neurons and astrocytes might be co-regulated early in development, potentially guided by sensory modality. Astrocytes in the thalamus are generated during embryonic development, with their emergence occurring around E17-E18—a timeline similar to that of astrogenesis in other brain regions, where astrocyte production typically begins around E18^36^. However, the precise timing of astrocyte generation in the thalamus relative to other brain regions remains unclear. These findings raise the possibility that astrocytes play a role in the early specification of thalamic circuits, though further research is needed to establish the direct connection between the sensory modality-specific identities of both mature neurons and astrocytes and their progenitors.

#### Toward a molecular logic of thalamic differentiation

In sum, advances in scRNA-seq have provided unprecedented insights into the distinct trajectories of thalamic progenitors and their postmitotic neurons, confirming that thalamic progenitor domains are specified early on and follow defined genetic programs to generate diverse nuclei, including the sensory modality-specific ones^16^. Complementary lineage barcoding, along with high-throughput omics, could further refine these findings by providing robust confirmation and potentially revealing additional complexities in these genetic lineage trajectories. Finally, the formation of sensory modality-specific thalamic nuclei is governed by both shared and unique genetic programs. The inherent flexibility within these pathways allows for cross-modal plasticity and adaptive reorganization, offering promising avenues for therapeutic interventions in sensory processing disorders.

## Mechanisms of sensory thalamocortical circuits development: Thalamus-derived signals

Beyond the early molecular determinants of thalamic nuclei specification, thalamocortical circuit formation and refinement are sequentially influenced by the emergence of spontaneous activity patterns, the so-called thalamic waves. Arising later in development, these waves drive activity-dependent modifications while occurring in parallel with transcriptional programs that regulate axon guidance and synaptic formation.

### Thalamic waves

**Fig. 3:**
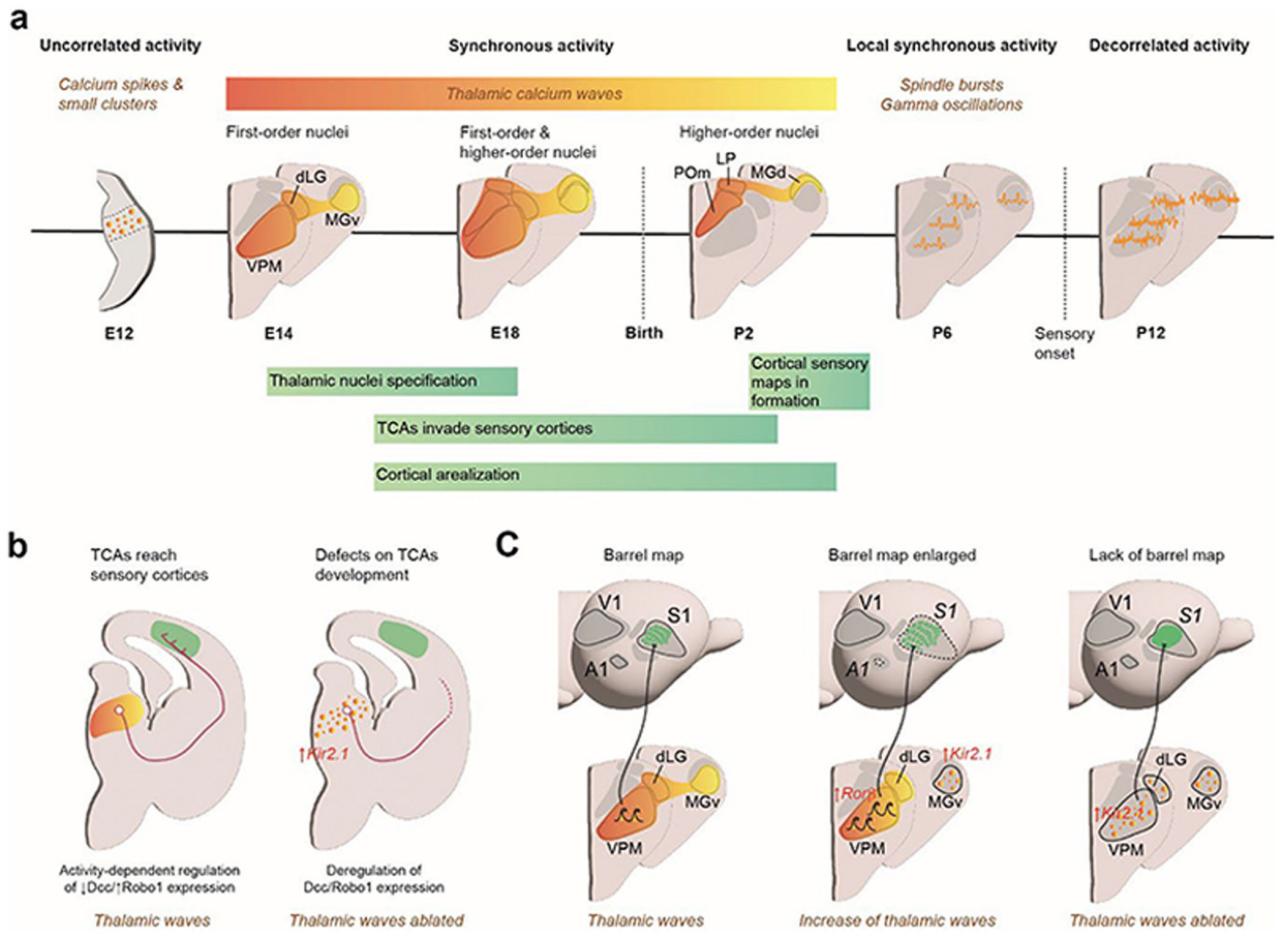
The role of thalamic waves in sensory cortical map development. **a**, Developmental timeline of thalamic activity. Thalamic spontaneous activity undergoes dynamic changes throughout embryonic and early postnatal development in mice. These changes occur in parallel with thalamic nuclei specification, cortical arealization, TCA pathfinding and formation of sensory maps in the cortex. The discrete activity patches shown in the early stages represent localized calcium spikes and small clusters of activity. These spatially restricted events are distinct from the broader thalamic calcium waves observed later in development. These calcium waves are characterized by synchronous activity that propagates sequentially across thalamic nuclei. The color gradient (from dark orange to light yellow) illustrates the temporal sequence of this propagation. Each wave typically unfolds over a timescale of several seconds. **b**, Regulation of Dcc and Robo1 expression in the thalamus. Under normal conditions, thalamic waves orchestrate a transition in gene expression during development, shifting from high Dcc and low Robo1 levels to low Dcc and high Robo1 levels. This regulation ensures the proper growth and targeting of TCAs toward sensory cortical areas. In the absence of thalamic waves, sparse and asynchronous activity disrupts this transition, impairing TCAs growth and navigation. **c**, Formation of barrel maps. During normal development (left), thalamic waves emerge around E14 and propagate through primary sensory nuclei, coinciding with thalamocortical axon migration to the cortex. In MGv-Kir transgenic mice (middle), where thalamic spontaneous activity is selectively silenced in the MGv nucleus is absent, spontaneous activity in the VPM nucleus increases, leading to an enlarged barrel map and a reduced auditory cortex. In the absence of thalamic waves (Th-Kir transgenic mice) (right), barrel formation is disrupted, even though TCA navigation may remain intact. Abbreviations: A1, primary auditory cortex; E, embryonic day; dLG, dorsal lateral geniculate nucleus; LP, lateral posterior nucleus; MGd, dorsal division of the medial geniculate body; MGv, ventral division of the medial geniculate body; P, postnatal day; POm, posterior medial nucleus; S1, primary somatosensory cortex; TCAs, thalamocortical axons; V1, primary visual cortex; VPM, ventral posterior medial nucleus.

Thalamic waves are spontaneous burst of propagating calcium activity mediated by gap junctions which are essential for establishing early thalamocortical connectivity. Thalamic spontaneous activity begins at the end of the second gestational week in mice with endogenous, uncorrelated activity (E12–E14). By E14, thalamic activity becomes synchronized, forming waves that propagate across prospective thalamic nuclei, initially engaging only FO nuclei and, by E18, also HO nuclei^37–39^. The frequency of thalamic waves decreases during perinatal stages, with somatosensory and auditory waves ceasing at birth, and visual waves ceasing around postnatal day (P) 2 in *ex vivo* slices^37^. Whether this pattern is consistent *in vivo* remains to be determined (Fig. 3a).

Synchronized activity facilitates synaptic refinement, aligns sensory inputs to cortical regions, and orchestrates activity-dependent developmental processes necessary for shaping thalamocortical circuitry. Experimental manipulations of these bursts of activity have revealed changes in gene expression related to the growth and branching of thalamocortical axons^37,40–45^. Specifically, thalamic waves regulate the extension of thalamocortical axons (TCAs) by modulating the expression of receptors such as Robo1 and Dcc, which act as brakes or as accelerators for axonal growth, respectively^42,44^. The precise temporal regulation of axon extension and cortical entry is critical for establishing functional thalamocortical circuitry. Aberrant spontaneous activity, leading to dysregulation of these gene expression profiles, compromise thalamocortical connectivity, and may ultimately result in deficits in cortical arealization and sensory processing. Thalamic waves also play a crucial role in regulating the size of cortical areas. Increased frequency of spontaneous thalamic waves has been shown to drive the expansion of the corresponding primary cortical regions by modulating the expression of the thalamic transcription factor Rorb^37^. Rorb finely tunes the complexity of axonal arborization within thalamocortical projections, specifically influencing axonal branching and length. This regulation occurs in a spontaneous activity-dependent manner, directly contributing to cortical enlargement and the structural organization of thalamocortical pathways (Fig. 3b).

A significant role of calcium activity and electrical synchronization lies in their capacity to facilitate synapse stabilization and pruning. During early developmental stages, an overabundance of synaptic connections is initially formed, which is later refined through activity-dependent mechanisms. Thalamic correlated activity orchestrates synchronized firing across neuronal populations, creating patterns that selectively strengthen synapses that establish appropriate connections while eliminating redundant or unnecessary ones^43,46,47^. Although the temporal dynamics of thalamic waves differ between FO and HO nuclei, direct evidence linking calcium activity patterns to the differential maturation of pruning across these nuclei remains limited. For instance, no significant differences were found in the timing of synaptic refinement between FO and HO nuclei in the thalamic reticular nucleus of newborn kittens, suggesting that pruning may occur concurrently despite differences in prenatal activity^48^. This dynamic process of synaptic pruning refines the functional architecture of thalamocortical circuits, ensuring that only the most functionally relevant connections are preserved, thereby optimizing sensory information processing and transmission^49,50^. Abnormal patterns of synchronized activity –whether due to genetic mutations, environmental factors, or neurodevelopmental disorders– can lead to aberrant synaptic connections in thalamocortical pathways, ultimately resulting in sensory deficits^51–53^, highlighting the importance of early correlated activity in circuit formation^43,54^.

A critical function of thalamic waves is their contribution to the formation of topographic maps. These waves synchronize neuronal firing patterns across the developing thalamus and cortex, aligning sensory inputs to their respective cortical areas. This arrangement is fundamental for generating sensory representations, such as the somatotopic map in the somatosensory cortex^45^. Antón-Bolaños and colleagues^45^ demonstrated how thalamic waves in the embryonic thalamus shape the development of cortical columns and the functional emergence of somatotopic maps. Through the temporal and spatial coordination of neuronal activity, thalamic waves ensure that sensory input is organized topographically, establishing the basis for precise perception and interaction with the environment (Fig. 3c).

Thalamic calcium waves fade by P2 as shown in brain slices^37^, however, their electrical correlate and temporal window *in vivo* are still unknown. *In vivo* extracellular recordings of early postnatal mice show that thalamic activity exhibits spontaneous, discontinuous bouts of gamma, spindle, or combined oscillations^55,56^. These oscillations, primarily driven by sensory organ inputs, can also be triggered by external stimuli but are not solely dependent on them. Crucially, these thalamic oscillations engage the cortex in a coherent manner, playing a vital role in establishing proper thalamocortical connections, as evidenced by the synchronized activity observed between somatosensory barreloids and their corresponding cortical barrels^57,58^. After the first postnatal week in mice, thalamic activity undergoes a significant transition, becoming more continuous and influenced by the animal’s behavioral state^59^. Consistently, the activity in the cortex becomes decorrelated also during the second postnatal week^60,61^. This transformation facilitates the integration of sensory driven neuronal activity, which becomes crucial for refining neural networks. Altogether, these processes shape the final formation and maturation of sensory maps enabling the establishment of functional neural circuits^62,63^.

### Thalamic molecular codes

Although the identity of thalamic neurons is determined by intrinsic genetic programs early on, the establishment and maintenance of thalamocortical circuits relies on the unfolding of a molecular program that, complementing the role of thalamic waves, regulates axon guidance, neuronal growth, and synaptic stabilization.

Transcription factors, for instance, are crucial regulators of thalamocortical development, controlling the expression of multiple genes involved in neuronal differentiation and circuit formation. This expression can be inherited from progenitors, as many transcription factors establish neuronal identity during early differentiation. However, emerging evidence suggests that thalamic activity can also influence transcriptional programs, potentially refining gene expression profiles in a nucleus-specific manner^42,44^. This dual regulation provides a crucial link between early genetic programs and activity-dependent mechanisms in shaping thalamocortical circuits. For instance, Gbx2 and Lhx2 play a regulatory role by orchestrating the genetic programs required for thalamic neuron identity, differentiation and connectivity^64–66^. The absence of Gbx2 disrupts the expression of LIM-domain transcription factors (Lhx2, Lhx9) and guidance receptors (Robo1 and Robo2), leading to TCA loss and failed cortical targeting^64^. Lhx2 further regulates the expression of Robo1 and Robo2, reinforcing the role of Lhx2 in establishing correct cortical connections^65^. This emphasizes the interplay between Gbx2 and Lhx2 in coordinating transcriptional and receptor codes to guide axonal connections and establish topographic patterns. Another key player in thalamocortical development is Foxp2. This transcription factor influences thalamic nuclei identity and axonal projection patterns. Disruptions in Foxp2 expression result in miswiring of thalamocortical axons, impairing sensory processing and cortical organization^67^.

Beyond transcription factors, molecular guidance cues further direct thalamic axon pathfinding, ensuring precise cortical connectivity. Among these, the ephrin/Eph receptor family is particularly important in guiding thalamic axons to their cortical targets, ensuring accurate sensory modality mapping. Ephrin-A5, expressed in cortical gradients and the medial thalamus, regulates thalamic neurite outgrowth, axonal trajectories, and terminal arborization, which are essential for forming precise sensory topography and functional synaptic connections^68–70^. Receptors for ephrin-A5, including EphA3, EphA4, and EphA5, display distinct expression profiles in the developing thalamus. The graded distribution of ephrin-A5 in the developing subplate and cortex, along with the expression of its receptors in the thalamus, emphasizes their role in the topographic mapping of thalamic axons to specific cortical regions^69,71^. EphA4, expressed in the lateral thalamus (visual and somatosensory nuclei), is particularly important for regulating axonal growth and pathfinding through interactions with ephrin-B3 and ephrin-A5^72,73^. Beyond axonal guidance, ephrin/Eph signaling contributes to synaptic stabilization, ensuring the robustness of sensory processing networks.

Once axons reach their appropriate cortical targets, further refinement is necessary to establish functional sensory maps. This process is heavily influenced by neurotrophic factors, including brain-derived neurotrophic factor (BDNF), which are involved in the development and fine-tuning of thalamocortical circuits^74^. By binding to its receptor TrkB, BDNF activates signaling pathways to promote the survival, differentiation and maturation of thalamic neurons while supporting axonal growth and synapse stabilization^75,76^. The influence of BDNF extends to activity-dependent plasticity, where it modulates the response of thalamic axons to environmental and sensory inputs. In the visual system, BDNF is essential for refining thalamocortical projections during the development of ocular dominance columns^75^. Similarly, Jiao and colleagues demonstrated that activity-driven BDNF expression is critical for forming barrel maps in the somatosensory cortex, as it links sensory input to changes in the barrel cortex, with disruptions preventing whisker activity from shaping GABAergic circuits^77^. BDNF and TrkB signaling contribute to sensory map organization and cortical structure, as their absence leads to disrupted thalamocortical connections, cortical degeneration, and deficits in synaptic plasticity during critical periods, underscoring their role in sensory network development^76,78^. Beyond sensory systems, alterations in BDNF signaling are associated with neurodevelopmental disorders, highlighting its broader significance in brain development and function^79^.

The establishment of sensory maps also relies on activity-dependent mechanisms, as neurotransmitters and their synaptic partners play a crucial role in shaping, refining, and stabilizing thalamocortical projections during postnatal development. Group I metabotropic glutamate receptors (mGluR1and mGluR5) are critical for cortical plasticity^80^ and the differentiation of cortical architecture shaped by thalamocortical axons. mGluR5 and its downstream effector, G-protein-coupled phosphodiesterase (Plcβ1), are essential for barrel development in the somatosensory cortex. Mice lacking mGluR5 exhibit normal row segregation of thalamocortical axons but fail to form these structures^81^. Interestingly, Plcβ1 deletion also disrupts barrel formation without altering thalamocortical patterning, suggesting that mGluR5-activated Plcβ1 signaling is crucial for barrel map emergence during the first postnatal week. The process governing the segregation of thalamic axons based on the peripheral pattern remains unclear. Additionally, serotonin (5-hydroxytryptamine, 5-HT) plays also a significant role in thalamocortical development by facilitating the initiation of synaptic interactions and the elaboration of axonal branching. Alterations in 5-HT levels lead to disruptions in thalamocortical patterning and cortical barrel formation^82,83^.

In addition to neurotransmission, the regulation of neuronal excitability by ion channels is essential for thalamocortical circuit refinement and sensory map formation. In thalamic neurons, channels such as T-type calcium channels (*CACNA1G*) influence oscillatory dynamics, modulating thalamic activity critical for TCAs development and sensory map formation^45,84,85^. Variations in ion channel expression, including potassium channels (Kir), which regulate action potential thresholds within thalamic neurons, might disrupt excitability and activity-dependent axonal branching and circuit refinement^45^.

Beyond genetic programs and activity-dependent mechanisms, epigenetic regulation ensures that thalamocortical circuits adapt to environmental influences, providing an additional layer of developmental control. Epigenetic mechanisms, including DNA methylation and histone acetylation, also control thalamic gene expression by modulating gene accessibility and responsiveness to environmental cues during critical developmental periods^86,87^. In particular, deacetylation of histone H4 is a necessary for barrel map formation in the somatosensory cortex. As demonstrated by Fetter-Pruneda and colleagues, premature deacetylation in birth-enucleated rats accelerates barrel development^88^. Conversely, inhibiting H4 deacetylation delays this process, restoring proper developmental timing and preventing abnormal barrel expansion. Another example of histone deacetylase involved in TCA branching is HDAC9 that interacts with TFs like MEF2 to regulate thalamocortical axonal growth during development^89^. These modifications allow thalamocortical circuits to adapt to diverse developmental contexts, ensuring precise sensory alignment even under variable conditions.

In sum, the combined influence of thalamic waves and gene expression, with the additional layer of epigenetic modulation, form an integrated system driving the development of thalamocortical circuits. These components collectively ensure that sensory information is organized, spatially aligned, and optimally refined for accurate perception and response, underlying the functional architecture of the sensory cortex.

## Navigation towards the cortex and early cortical interactions

### Thalamic axonal pathfinding

**Fig. 4:**
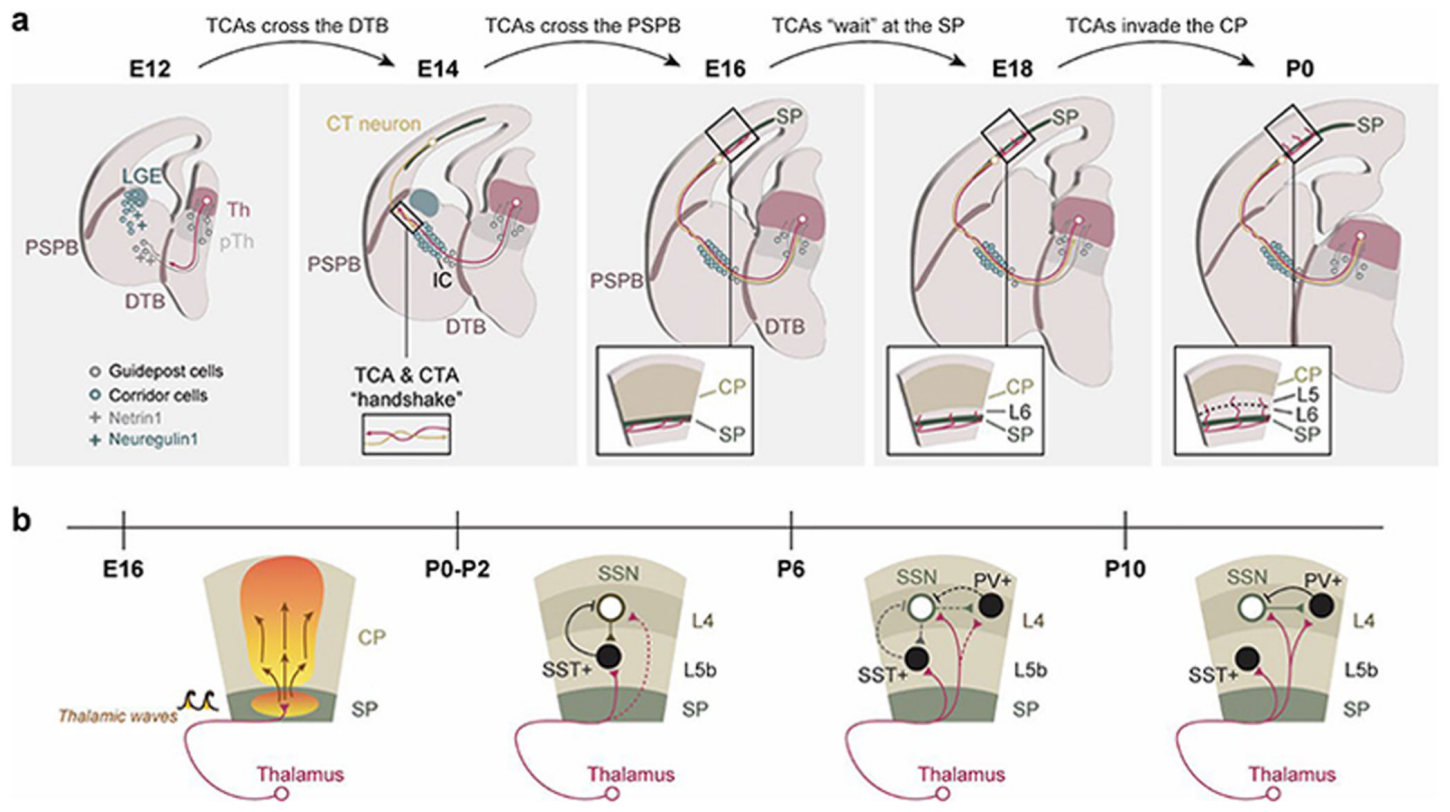
Thalamocortical axon navigation through the ventral telencephalon towards the cortex. **a**, Shortly after they are born, thalamic neurons extend their axons and cross the DTB with the help of guidepost cells, signaling molecules (like Netrin1 and Neuregulin1) and corridor cells. At around E14 in mice, thalamocortical axons (TCAs) intermingle with corticothalamic axons (CTAs), using them as scaffolds to cross the pallium-subpallium boundary (PSPB). After crossing the PSPB, axons wait at the subplate (SP) at the appropriate cortical region, until they invade the cortical plate around birth. **b**, Sequential contact of thalamic axons with its cortical targets. Initially (E16-P0), thalamic axons displaying thalamic waves contact the subplate network, which transmits this activity dorsally to the cortical plate. By P4, thalamic axons establish synapses with L5b somatostatin-positive interneurons, which establish a transient (P4-P9) reciprocal connection with layer 4 spiny stellate neurons. This transient circuit is essential for the formation of local circuitry, including the feedforward inhibition by parvalbumin-positive interneurons. Finally (P10-P15, the thalamic connectivity with layer 4 spiny stellate, as well as layer 4 local connectivity, is established. CP, cortical plate; CT, corticothalamic; dLG, dorsal lateral geniculate nucleus; dSC, deep superior colliculus; DTB, diencephalon-telencephalon boundary; DTh, dorsal thalamus; IC, internal capsule; L, layer; LGE, lateral ganglionic eminence; P, postnatal stage ; pTh, prethalamus; PV, parvalbumin-positive interneuron; S1, primary somatosensory cortex; SP, subplate; sSC, superficial superior colliculus; SSN, spiny stellate neuron; SST, somatostatin-positive interneuron.

Sensory function is reliant on a reciprocal thalamus-cortex connectivity that develops early on during development. Although cortical and thalamic neurons are born around the same time in mice (E11-E17 in the cortex^90^, E12-E15 in the thalamus^28^, they develop in parallel until their first functional contact right before birth. This connectivity is initiated by TCAs, which traverse the diencephalon-telencephalon boundary (DTB) first towards the internal capsule and later go through the pallium-subpallium boundary to reach the first cortical target: the subplate (Fig. 4a).

In the earliest stages of thalamic development, between E12 and E14, thalamocortical axons extend rostrally through the prethalamus and encounter their first challenge: crossing the DTB to reach the ventral telencephalon. At these earliest stages, cells located in the prethalamus have been shown to project caudally to the thalamus. These cells have been referred to as “guideposts”, as their projections may act as scaffolds to guide TCAs to cross the DTB and enter the internal capsule^91–94^. These cells also provide positional cues, like Netrin1, which is also expressed by cells in the internal capsule and acts as a chemoattractant for TCAs^95^ (Fig. 4a). Other domains of gene expression provide migrating cues to the coursing axons during this first stage of the journey. For example, *Ascl1* (*Mash-1*) is expressed in the ventricular zones of the prethalamus and the ventral telencephalon and in *Ascl1*-deficient mice, TCA fail to project beyond the DTB^96^. Deletion of *Ebf1*, which is mostly expressed in the ganglionic eminences during development^97^, causes a shift in thalamocortical pathfinding towards more caudal regions of the cortex, and also results in some thalamic axons abnormally projecting towards the amygdala^98^. *Dlx1* and *Dlx2* are expressed in the prethalamus and the basal ganglia^19^ and in *Dlx1/2*-/- mutants most of the thalamic axons fail to reach the cortex and those that do show a shifted topography^98^. These cues are specific for thalamic navigation and not merely related to their position along the TCA path, since, for example, deletion of *Nkx2*.*1*, normally expressed in the basal telencephalon, severely affects layer 5 corticofugal projections but spares TCA pathfinding^99^. Finally, an intriguing discovery unveiled that a population of tangentially migrating neurons, born in the nearby lateral ganglionic eminence (LGE), provide a bridge for the thalamocortical (TC) axon pathfinding through the internal capsule. The so-called corridor cells create this permissive corridor for TCAs through the expression of membrane-bound isoforms of Neuregulin1 and are a *sine qua non* condition for proper TCA navigation^100^ (Fig. 4a).

The pallium-subpallium boundary (PSPB) is a particularly non-permissive barrier, formed by a prominent radial glial fascicle^101–103^ and the densely packed cells of the lateral cortical stream, a mix of pallial and subpallial progenitors that migrate towards the basal telencephalon^104^, thus constituting a mechanical barrier that hinders TCA navigation. The cortical preplate axons cross the PSPB earlier than TCAs do, around E13-E14 in rodents^105,106^. According to the “handshake hypothesis”, TCAs then intermingle and form fascicles with cortical axons, using them as a scaffold to cross the PSPB and reach their corresponding cortical region^93,107^ (Fig. 4a). Mutant mice where cortex-specific genes, such as *Reelin*^107^, *Tbr1, Pax6*^108,109^ or *Emx2*^110^ are deleted portray defects in thalamocortical guidance already at the internal capsule, supporting this hypothesis. Similarly, loss of *Gbx2*, expressed by thalamic cells, leads to complete loss thalamocortical development, but also alters corticofugal pathfinding^108^.

After crossing the PSPB, TCAs enter the subplate around E15 in rodents and take a few days before entering the cortical plate (CP)^54^ (Fig. 4a). Subplate neurons are born early and mature before their cortical counterparts, but most of them disappear by programmed cell death^111,112^. Although transient, their presence is required for appropriate thalamocortical innervation^113–115^. Within the subplate layer, thalamic axons give off transient collaterals^116,117^ and establish glutamatergic synapses with subplate neurons^118–121^. Subplate neurons thus receive spontaneous synaptic input, and this neural activity participates in the pathfinding process, since blocking voltage-gated sodium channels causes abnormal target selection of thalamocortical axons^122^. In a recent study, Doyle and colleagues demonstrated that cortical deletion of *Arid1a* led to disrupted thalamocortical ingrowth, due to impaired PSPB crossing and overall pathfinding, resulting in fewer and disorganized barrels in the somatosensory cortex. When this cortical deletion spared the subplate neurons, however, TCA targeting is not affected, and barrels develop normally^123^. After the “waiting period” at the subplate, shortly before birth, thalamocortical axons invade the CP until they reach layer 4 (Fig. 4a). This arrest is thought to be mediated by an inhibitory or “stop” signal that reduces the growth rate of thalamic axons, allowing for proper layer 4 targeting^124–126^.

### Cortical control of thalamic ingrowth

In the cortex, intrinsic control of arealization is evidenced by the graded, or restricted, expression of genes that encode TFs, axon guidance ligands and receptors or cell adhesion molecules^69,127–129^. These genes are expressed in the ventricular zone or the CP prior to the arrival of TCAs^130^ and presumably influence the arrangement of thalamic axons upon arrival to the CP in an area-specific manner. *Emx2* and *Pax6*, for instance, show complimentary gradients of expression in the developing cortex^131,132^, imparting caudomedial and rostrolateral identities to cortical neurons, respectively. In *Emx2*-deficient mice, the rostral-lateral areas are expanded while caudomedial areas are reduced, and the opposite occurs in *Pax6* mutant mice^133,134^. The shift in arealization in *Emx2* mutant mice is matched by a shifted thalamocortical innervation, while in the *Pax6* mutants, thalamocortical axons fail to reach the cortex^133^. This total lack of TC innervation in *Pax6* mutants is probably not due to cortical defects but to anatomical alterations in the DTB^109,135,136^, as it does not happen when the gene is mutated after the formation of this interface^135^. Indeed, when *Pax6* was deleted only in the cortex, TCA topography was not affected. Conversely, when *Pax6* deletion affects the diencephalon, TCAs present topographic errors at the earliest stages of navigation^137^. COUP-TF1 (also called Nr2f1) is a transcription factor expressed by the subplate and CP in a high caudal to low rostral gradient^138^. Mice deficient for COUP-TF1 show reduced thalamocortical innervation and the ventral posterior nucleus of the thalamus, which projects to S1 in normal conditions, sends projections to the caudal cortex, where V1 eventually develops^139^. These results are hard to interpret, as the phenotype could be since COUP-TF1 is also highly expressed in the thalamus itself^138,140^.

Graded gene expression in the developing cortex is set by patterning centers distributed along the neuraxis, which produce signaling molecules like fibroblast growth factor (Fgf) 8, expressed in the anterior neural ridge, Sonic hedgehog (Shh) produced in the ventral telencephalon and bone morphogenetic proteins (Bmps) and Wnts, expressed in the cortical hem^141–145^. In knock-out mice for Gli2, an intermediate transcription factor of the Shh signaling pathway, TCAs are misrouted towards the hypothalamus and the anterior commissure or stop at the LGE^146^. Increasing endogenous levels of Fgf8 *in utero* results in the expansion of frontal domains at the expense of caudal domains, as demonstrated by the shift in ephrin-A5 expression towards the posterior pole. By contrast, decreasing Fgf8 results in cortical area boundaries shifting anteriorly^147^. Intriguingly, ectopic expression in the caudal cortex led to a partial duplication of the barrel field^147^. Since barrel formation depends on the orderly arrangement of TCA, these results suggest that Fgf8 influences TC targeting of cortical areas by inducing a thalamic reorganization, reflecting the bidirectionality of this circuit. This was partly demonstrated by Shimogori and Grove^148^, when they showed that TCAs faithfully track the shifts in cortical areas induced by the misexpression of Fgf8, and that they also innervate the duplicate barrel field caused by an ectopic source of Fgf8. More strikingly, depending on the timing of misexpression of Fgf8, the regional identity of the subplate and CP could be in or out of registry. In fact, Fgf8 overexpression at E11.5 or later, caused a caudal shift in the CP but not in the subplate. As a result, the thalamic axons innervated layer 4 of the shifted S1 but not the deeper layers^148^. These results highlight the differential roles in TC axon guidance of subplate and CP, suggesting that the subplate regulates the laminar arrangement of thalamocortical innervation. Future experiments should further explore to what extent these cortical manipulations provoke changes in gene expression in the thalamus itself. Finally, IPCs in the vicinity of ingrowing TCAs can also influence their intracortical navigation. Indeed, Abe and colleagues demonstrated that C-X-C motif chemokine 12 (CXCL12) released by IPCs guides TCAs, which show strong expression of its receptor CXCR4, as IPC-specific ablation of *Cxcl12* resulted in attenuated TC ingrowth^149^.

The molecular processes regulating thalamocortical targeting seem to be very robust. For example, despite profound distortions in cortical neuron organization in the Reeler mouse, including the inversion of cortical layers, the thalamic nucleus-to-cortical area relationship and the topographic organization of axons are for the most part preserved^150^. Similarly, while in sensory deprivation scenarios profound reorganizations and adaptations of sensory pathways occur (see “Early plasticity of thalamocortical circuits” section), the appropriate thalamic nucleus-to-cortical area synaptic matching is maintained^151^.

### Thalamic influence on cortical development

Great progress has been made in understanding the intrinsic mechanisms acting at the level of the cortical neuroepithelium in the neocortical acquisition of cell diversity and arealization^90^. In Gbx2 mutants, where thalamic differentiation is perturbed, the broad subdivisions of the cortex are preserved, suggesting a primary role of local, cortical gene expression in the acquisition of areal identity^130^. However, these mice do not live past P0, leaving the effect of thalamic perturbation on cortical differentiation hard to assess. On the other hand, the early arrival of thalamic axons at the subplate and CP, when the cells of both are still differentiating, strongly suggests that thalamic influence must have a primary role in cortical maturation. In fact, already at E18, a peripheral tactile stimulus is able to trigger a cortical calcium transient in the somatosensory cortex, which means that thalamic activation propagates to and excites cortical neurons^45,151^, potentially influencing their maturation. Some recent findings have deepened our understanding of the mechanisms through which the thalamus could exert its cortical influence. As discussed above, prenatal thalamic waves have a critical impact in thalamocortical development by regulating cortical area size^37^ and the formation of the cortical somatotopic map^45^. At initial stages, thalamic activation of the cortex starts in the subplate, where TCAs form functional synapses^111^. This activation triggers local neural networks coupled by gap junctions and chemical synapses^152,153^ that then propagate to the CP above^45^ (Fig. 4b). This transient connectivity of TCAs with subplate cells seems to be fundamental for appropriate cortical development. Indeed, neurons in the subplate form local networks of recurrent connectivity^152^ which amplify the afferent thalamic connectivity^154,155^, thus influencing the emergence of features like ocular dominance columns or the maturation of cortical inhibition^156^. Thalamic axons form transient connections with somatostatin interneurons in layer 5b^157^, which in turn temporarily connect to L4 spiny stellate neurons (SSNs)^158^. This transient loop is essential for the timely formation of TC synapses onto L4 SSNs, as well as for the local L4 circuits (Fig. 4b). Failure to eliminate the transient TC-SSN connection, a process mediated by metabotropic glutamate receptor signaling, can alter exploratory behavior in adult mice^159^. When L4 SSNs receive thalamic contact, they start orienting their arbors towards the presynaptic terminals, in a process that is mediated by NMDA receptor signaling^160^. Finally, thalamic axons can also modulate the connectivity patterns of their target neurons. For example, during development, layer 4 neurons form transient callosal projections to the contralateral hemisphere, and these exuberant axons get pruned in a thalamic input-dependent manner^161^. Thalamic input onto layer 1 interneurons during development is also essential for the appropriate formation of top-down projections from the higher-order anterior cingulate cortex to these interneurons^162^.

Whereas intrinsic genetic factors set broad subdivisions in the cortex, thalamic axons seem to control the fine distinctions between first-order and higher-order areas within a given sensory modality. For instance, in the visual system, geniculocortical input drives genetic differences between first-order and higher-order visual cortices^163,164^. In the somatosensory system, ablation of the first-order VPM nucleus of the thalamus causes the rewiring of the higher-order POm nucleus to L4 neurons, which become molecularly and functionally respecified to acquire typical POm-target features^165^. Other ways that thalamic input has been shown to modulate cortex development are through regulation of cortical lamination^166^ and dendritic orientation^167^. In the somatosensory cortex, barrel hollows, the main recipient of VPM input, are populated by spiny stellate neurons, which display asymmetric dendritic arbors directed towards the axon terminals. Conversely, barrel septa are populated by pyramidal neurons with distinct connectivity from their hollow counterparts. In a recent study, spatial transcriptomics revealed reciprocal patterns of gene expression in barrel hollows versus septa. Young and colleagues found that the default state is that of the septumpyramidal neuron identity, which requires TCA input to be subdivided into hollowstellate and septumpyramid subdivisions^168^. Indeed, the loss of TCA innervation resulted in the downregulation of several hollow-related genes and prevented the acquisition of spiny stellate fate, suggesting an instructive role of thalamic input in the acquisition of cell diversity through the upregulation of specific sets of genes^168^. Altogether, these results suggest that, although intrinsic molecular cues at the neuroepithelium set out broad differences across areas in the cortex, thalamic input leads to final specification of cortical neurons upon arrival. These interactions between intrinsic and extrinsic mechanisms may be even more complex than previously recognized, as it has been demonstrated that cortical neurons that reside in the same cortical area but derive from distinct progenitors differ in the thalamic inputs they receive^169,170^.

## Sub-thalamic influence in the development of sensory modality thalamocortical circuits

The thalamus is considered the main relay station for sensory inputs to reach the cortex. Thus, the auditory nucleus of the thalamus, the MGv, receives bilateral input from the cochlea through intermediate structures of the brainstem and midbrain; the visual dLG receives direct retinal input, and the somatosensory VPM nucleus receives input from the principal trigeminal nucleus (PrV), which in turn receives peripheral tactile stimuli. Already at embryonic stages, these nuclei receive inputs from primary sensory neurons, which display spontaneous bursts of activity^39^. For instance, in the sensorimotor system, spontaneous body movement called twitches cause activation of the matching somatotopic area in the sensory spinal cord^171,172^. In the cochlea, periodic bursts of spontaneous activity are generated spontaneously from embryonic stages^173–175^, way before the opening of the ear canal during the second postnatal week^176^. Retinal waves are probably the most-studied form of spontaneous activity and are generated in three consecutive stages, with stage 1 waves starting embryonically, and stage 3 waves extending to the time of eye opening, at around P12 in mice^177^. At these early stages, peripheral-to-central pathways are already established and thus these forms of spontaneous activity get transmitted upstream. In fact, at E18 in mice, when thalamic axons start invading the cortex, sensory connectivity is already established and a tactile stimulus in the whisker pad triggers a functional response in the contralateral barrel cortex^45,151^. Similarly, although the retina is not responsive to light at this age, spontaneous retinal waves get transmitted to the cortex before birth^151^. Thus, spontaneous activity of the sensory organs has the potential to ultimately influence thalamocortical targeting.

The role of peripheral input onto the formation of sensory circuits has been extensively studied by means of sensory early deprivation^178^. For example, altering retinal waves in the early postnatal brain has been shown to disrupt the precise retinotopy of geniculo-cortical maps^179,180^, Additionally, induced early blindness results in reduced size of the dLG and its target, V1^181–186^, whereas non-deprived areas, like the somatosensory cortex, expand^37,184^. The expansion of somatosensory areas was shown to be mediated by thalamic waves, as embryonic bilateral enucleation results in increased frequency of these waves in the VPM^37^. In addition, HO visual areas expand^187^ in response to visual deprivation, and cross-modal recruitment of visual circuits takes place (see following section). However, the geniculocortical projection is for the most part present and specific to V1. Even when visual deprivation is done embryonically, before retinal axons reach the dLG, and when the size of dLG and V1 is more strongly reduced, the geniculocortical connection appropriately targets V1^37,151^. In a seminal study by Sur and colleagues, retinal axons were forced to project to the primary auditory nucleus of the thalamus, the medial geniculate nucleus. Consistently, cells in the auditory cortex were found to respond to visual stimuli, demonstrating that the appropriate MGv-to-A1 projection remains unchanged^188^.

**Fig. 5:**
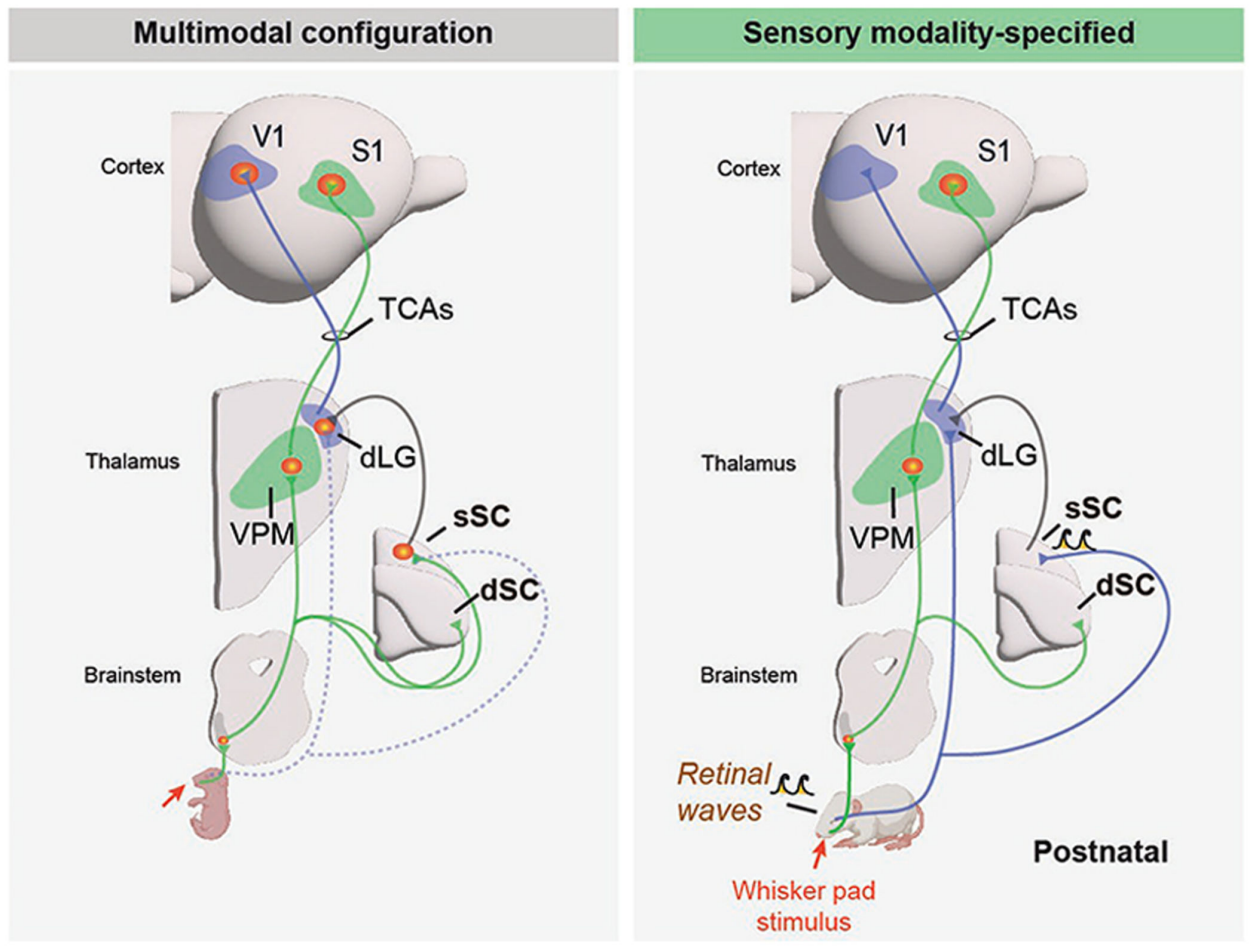
Developmental switch from multimodal to unimodal configuration in sensory circuits. Left. At embryonic stages, in addition to the expected activation of S1 through the activation of the VPM of the thalamus, a whisker pad stimulus reaches the superficial layer of the superior colliculus, which projects to the dLGN, which in turn projects to V1. Thus, a peripheral tactile stimulus triggers a multimodal response in the cortex, involving both developing S1 and V1. Right. Around birth, the earliest form of retinal waves targets the superficial layer of the superior colliculus, displacing somatosensory inputs. This reconfiguration of superior colliculus circuits, prompts sensory cortices to become specific to their corresponding sensory modality input at postnatal stages. Orange ovals represent neuronal activation triggered by a whisker pad stimulus at the different somatosensory (green) and visual (blue) stations. Abbreviations: dLG, dorsal lateral geniculate nucleus; S1, primary somatosensory cortex; dSC, deep superior colliculus; sSC, superficial superior colliculus; TCAs, thalamocortical axons; V1, primary visual cortex, VPM, ventral posterior medial nucleus. Mouse images were created in BioRender. Martini, F. (2025) https://BioRender.com/m24d847.

Altogether, these results further suggest that the intrinsic molecular mechanisms regulating thalamocortical axon pathfinding are remarkably robust and are not easily modified by peripheral input. However, they suggest that the sensory modality assigned to given cortical territory is determined by the type of peripheral input that the thalamic nucleus projecting to that cortex receives. This hypothesis was not proved until recently, when Guillamon-Vivancos and colleagues showed that, in the embryo, a tactile stimulus recruits the visual cortex through the activation of the dLG^151^. Upon the arrival of stage I retinal waves, V1 specializes in interpreting retinal activity and vision is ascribed to this cortical territory, whereas a tactile stimulus solely triggers an S1 response. In fact, blocking stage I retinal waves transiently or removing retinal projections in embryos impairs the timely developmental segregation of the somatosensory and visual modalities. Intriguingly, this work reveals that the segregation of sensory modalities does not directly occur at the thalamus, but at a retino-recipient structure that sends direct projections to the visual thalamus: the superior colliculus (SC). In the earliest, multimodal period, somatosensory inputs to the SC trigger the tectogenicular projection, activating the dLG and subsequently V1. At birth, and through the mediation of retinal waves, a reorganization of intra-collicular circuits mediates the segregation of sensory modalities, and the retino-colliculo-geniculate pathway is definitively established^151^. This study shows that, during development, the SC acts as a gatekeeper of sensory input that determines the sensory modality of thalamic nuclei and, consequently, of the cortices they project to (Fig. 5).

## Early plasticity of thalamocortical circuits: Intra-modal and cross-modal

The plasticity of thalamocortical circuits during early development is a fundamental process that underlies the brain’s ability to adapt and refine sensory information processing. This plasticity can be classified into intra-modal plasticity, which involves changes within a single sensory modality, and cross-modal plasticity, which involves interactions between different sensory modalities. Both forms are underpinned by a combination of activity-dependent processes, molecular signaling, and critical period dynamics, highlighting the intricate adaptability of the thalamocortical system during development. The timing and duration of critical windows vary across sensory modalities. The visual system exhibits a well-defined critical period for ocular dominance plasticity^189,190^. Similarly, the somatosensory and auditory systems each undergo distinct, well-characterized critical periods, with some degree of overlap in their sensitive phases, though their specific timelines and mechanisms of closure differ^191–193^. These windows dictate the extent of intra- and cross-modal plasticity, with early disruptions leading to long-lasting cortical reorganization^194^.

Additionally, associative cortical areas, which integrate multimodal sensory information, may facilitate cross-modal interactions via HO thalamic nuclei, enabling functional compensation following sensory loss, as observed in humans^195^. Understanding the interplay between thalamic and cortical mechanisms during these sensitive periods provides insights into how sensory circuits develop and reorganize in response to sensory deprivation.

### Intra-modal plasticity

**Fig. 6:**
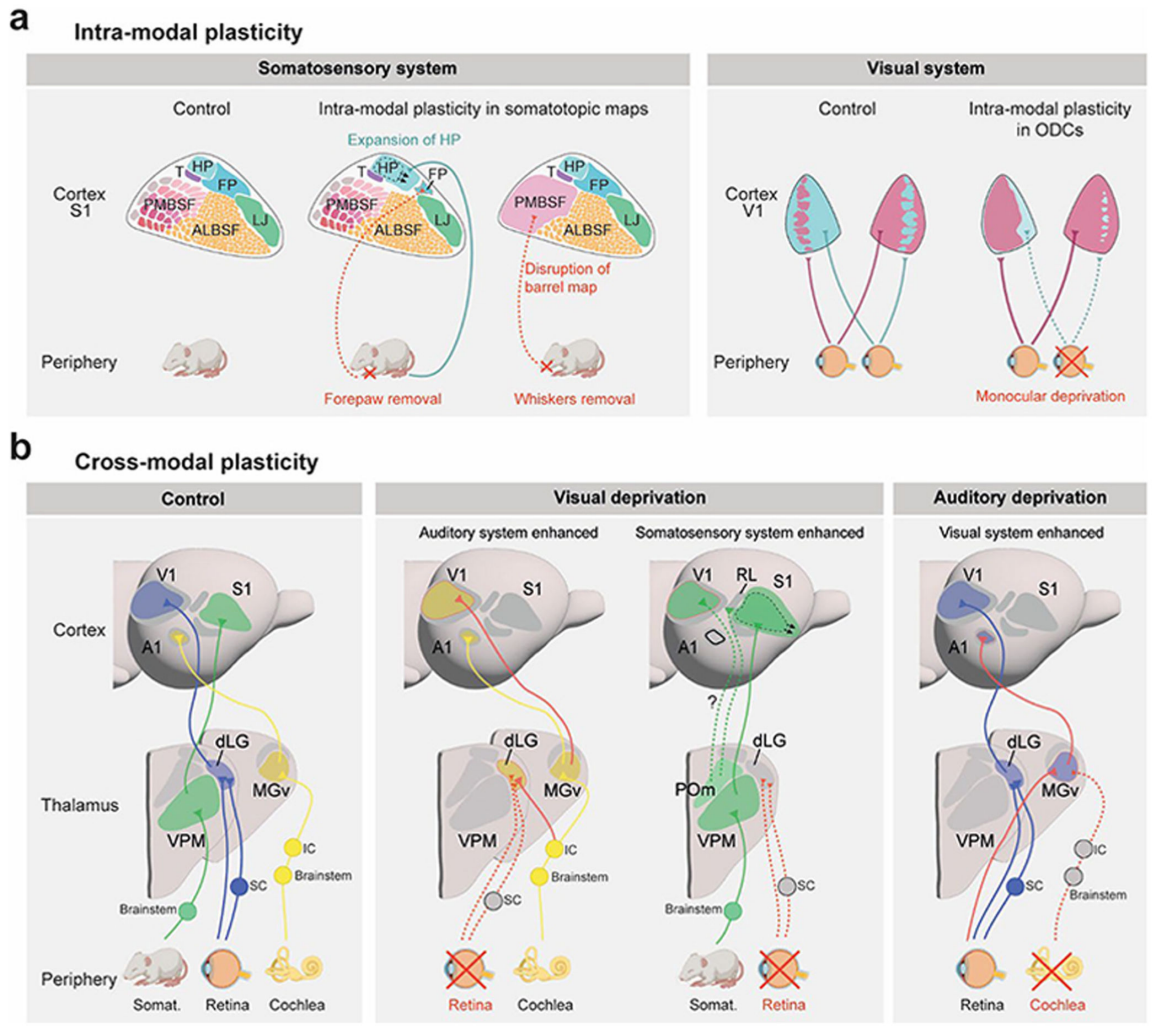
Thalamocortical plasticity. **a**, Intra-modal plasticity comprises the strengthening or remodeling of connections targeting their primary cortical area within the same sensory modality. Left, examples from the somatosensory system show that after forepaw removal at birth, the hindpaw cortical representation (HP) expands while the forepaw map (FP) diminishes. The second example represents whisker removal at P0 leading to the loss of barrel representations in the PMBSF. Right, in the visual system, monocular deprivation disrupts ocular dominance columns (OCDs), increasing the cortical area occupied by the intact eye through contralateral and ipsilateral projections. Dashed blue lines illustrate the loss of retinal axons from the deprived eye. **b**, Cross-modal plasticity involves the rewiring of one sensory modality in response to the deprivation of another. This schematic uses the mouse thalamocortical system as a representative model to illustrate key findings across various animal species. The axonal rewiring depicted highlights neural adaptations observed not only in mice but also in other models, demonstrating the diverse mechanisms that underlie sensory reorganization. Compared with control (left), visual deprivation (middle) enhances auditory and somatosensory systems. In the first example, in naturally blind mole rats, auditory system enhancement can occur through rewiring of IC axons from the MGv (in the control condition) to the dLG and the redirection, in opossums enucleated at birth, of MGv axons to the V1. Similarly, somatosensory enhancement involves the enlargement of the S1 in mice enucleated at birth, and rerouting of POm axons to RL or V1, in sighted mice. Right, after auditory deprivation, the visual system compensates by rerouting retinal axons to the MGv, allowing the A1 to process visual information in neonatal deaf ferrets and transgenic mice with IC ablation. Dashed red lines represent lost peripheral connections due to sensory deprivation or removal, while continuous lines indicate existing or newly stablished connections. Dashed green lines with question mark represent hypothesized newly stablished connections resulting from sensory deprivation. Blue, green and yellow lines represent axons transmitting visual, somatosensory and auditory information respectively. Continuous red lines represent axonal rewiring transmitting different sensory modality information. In the visual and auditory deprivation panels, only the connections associated with the enhanced sensory modality are depicted, while typical control connections retained in the deprivation models are not shown. Abbreviations: A1, primary auditory cortex; ALBSF, anterior lateral barrel subfield; dLG, dorsal lateral geniculate nucleus; IC, inferior colliculus; LJ, lower jaw; MGd, dorsal division of the medial geniculate body; MGv, ventral division of the medial geniculate body; ODCs, ocular dominance columns; PMBSF, posterior medial barrel subfield; POm, posterior medial nucleus; RL, rostrolateral; S1, primary somatosensory cortex; SC, superior colliculus; T, trunk; V1, primary visual cortex; VPM, ventral posterior medial nucleus. Mouse, retina and cochlea images were created in BioRender. Martini, F. (2025) https://BioRender.com/m24d847.

Intra-modal plasticity is essential for the development and refinement of sensory maps in the cortex, enabling precise representation of sensory stimuli. This process is driven by activity-dependent synaptic refinement where sensory inputs play a key role in sculpting these maps. In the somatosensory system, for instance, the barrel cortex provides a striking example, with sensory input from whiskers during early postnatal development organizing TCAs into distinct cortical barrels. Woolsey and Van der Loos^196^ first described the barrel cortex as a somatotopic representation of whiskers, providing a structural framework for studying sensory-driven plasticity. Subsequent work by Belford and Killackey^197^ and Killackey and Dawson^198^ demonstrated that early sensory deprivation (at P0), such as whisker or forelimb removal, alters somatosensory maps representation and thalamocortical connectivity (Fig. 6a). Woolsey and colleagues^199^ and Killackey and colleagues^200^ further revealed how thalamic inputs influence cortical map formation through activity dependent mechanisms, showing that competition among inputs refines synaptic connections to create sensory representations. More recently, Renier and colleagues^201^, focused on how sensory input influences the development and organization of sensory maps in the cortex, particularly in the barrel cortex. In this case, the altered sensory input in their mutant mice—specifically misrouting of trigemino-thalamic inputs—resulted in changes to the whisker map representation in the barrel cortex. Although the mutant mice showed the development of a bilateral map rather than the typical unilateral map, their study emphasizes how sensory inputs are critical for the organization of cortical representations, which is a key concept in intra-modal plasticity. Expanding on this concept, a recent study by Aníbal-Martínez and colleagues revealed that the temporal window for intra-modal reorganization of somatosensory maps is restricted to embryonic development in mice^194^. Their findings showed that deprivation of whisker input during the embryonic stage (E14) led to a permanent reorganization of the barrel field territories, whereas similar deprivation postnatally (P0) did not induce these changes. This study underscores the existence of a critical period during prenatal development for defining somatosensory cortical architecture and map resolution.

The critical period is a developmental window during which sensory input profoundly shapes neural circuits. Whisker damage during early postnatal stages disrupts cortical barrel organization, while similar manipulations at later stages result in minimal changes, emphasizing the temporal sensitivity of this period^202^. This heightened plasticity, spanning from embryonic development to P4, coincides with the ongoing formation and refinement of thalamocortical connections in the somatosensory system^193^. Similar findings in the visual system, such as the classical studies by Hubel and Wiesel^203^, demonstrated that depriving one eye during the critical period leads to permanent deficits in ocular dominance column formation and visual acuity, illustrating how monocular deprivation affects neural competition and cortical representation^204,205^. The visual system exhibits a delayed critical period for ocular dominance plasticity, ranging from embryonic stages to P15-P17^190^. Cabelli and colleagues^75^ demonstrated that during the critical period for ocular dominance formation, thalamocortical projections undergo substantial refinement, driven by sensory activity and the modulatory effects of BDNF signaling.

Neurotrophic factors such as BDNF and nerve growth factor (NGF) are pivotal in intra-modal plasticity, modulating synaptic strength, axonal growth and circuit refinement^76,79^. Synaptic plasticity mechanisms, including long-term potentiation (LTP) and long-term depression (LTD), further refine thalamocortical circuits by enhancing or weakening synapses in response to activity patterns regulated by molecular players like AMPA and NMDA receptors^206–208^. Critical periods for plasticity depend on reliable input to circuits, requiring precise coordination between excitatory and inhibitory networks. In particular, GABAergic signaling is crucial for initiating critical periods by creating the necessary balance for plasticity to occur^190,209^. These findings underscore the interplay of activity-dependent mechanisms and neurotrophic signals to ensure that sensory maps align with environmental inputs, laying the foundation for accurate sensory function throughout life.

### Cross-modal plasticity

Blind humans exhibit enhanced capacities in auditory, olfactory, and somatosensory discrimination due to compensatory neuroplasticity following to sensory deprivation^210–212^. Similarly, deaf individuals show superior visual discrimination abilities as supported by studies demonstrating increased activation in visual and restructured auditory regions^213–215^. Congenitally deaf individuals also exhibit improved tactile discrimination, likely associated with the rewiring of the somatosensory system, further reflecting cross-modal plasticity^216,217^. These findings underscore the brain’s remarkable capacity to adapt to sensory loss by redistributing sensory processing resources across modalities—through the rewiring of circuits, the strengthening of existing connections, and the weakening or repurposing of others to optimize neural processing.

Although traditionally thought to rely solely on sensory experience, cross-modal plasticity can occur before sensory inputs emerge, as shown in animal models deprived at early stages, before sensory onset^37,218,219^. These pre-sensory adaptations influence thalamocortical network maturation and provide a foundation for experience-driven changes. Understanding these mechanisms might help develop future interventions that harness the brain’s adaptive capabilities to support sensory impairments.

In enucleated animals (blind models), significant reorganization is observed. For instance, visually deprived rodents and kittens exhibit activation of the presumptive visual cortex by auditory and somatosensory stimuli^178,220,221^. This plasticity enhances tactile sensitivity, such as in whisker-related barrel cortex regions, with expanded representations and improved sensory responses^88,222,223^. Similarly, naturally blind mole rats demonstrate auditory activation of both the visual thalamus and visual cortex due to rerouted auditory connections, reflecting profound neural reorganization^220,224^. Indeed, in these animals the dLG is unusually innervated by the inferior colliculus (IC), the auditory relay preceding the thalamus, which also projects to the visual cortex^225^(Fig. 6b). Moreover, in opossums enucleated at birth, the auditory nucleus of the thalamus sends atypical projections to V1^226^. In mice enucleated at birth, changes in ephrin-A5 expression have been linked with significant alterations in cortical arealization^182^. Ephrin-A5 appears to mediate compensatory adaptations in shaping somatosensory-visual boundary in the neocortex when visual input is absent. In addition, embryonic enucleation leads to an increase in the barrel size, accompanied by an increase in the frequency of thalamic calcium waves and changes in *Rorb* expression in the VPM, contributing to increased complexity of TCA terminals^37^. Thalamic calcium waves are thought to facilitate cross-modal plasticity by coordinating gene expression patterns that shape the size of primary cortical areas before sensory experience^37^. These waves enable different sensory modalities to communicate and reorganize to adjust sensory maps. Furthermore, alternative unexplored pathways could mediate cross-modal plasticity that transfers somatosensory information into the visual circuits. In sighted mice, connections have been identified between the HO somatosensory nucleus of the thalamus POm and V1, as well as between the POm and HO visuo-tactile areas^227,228^. These findings suggest that early visual deprivation can lead to significant reorganization of subcortical pathways, allowing for the rerouting of somatosensory information.

In models of hearing deprivation, cross-modal plasticity involves the auditory cortex being co-opted for visual processing. For example, in neonatal deaf ferrets, retinal axons were experimentally rerouted to the medial geniculate nucleus (MG), leading to visual input being processed in the auditory cortex. This rewiring allowed the auditory cortex to mimic visual cortical functions, such as orientation and motion tuning^229^. Additionally, transgenic mice have enabled the creation of models with significantly altered thalamic sensory input. For instance, the abnormal retinal innervation of the MG following IC ablation is more pronounced in mice lacking ephrin-A2/A5 receptors, compared to wild-type mice^230^. This suggests that ephrin-A gradients play a role in patterning these rewired visual projections.

Collectively, these examples of cross-modal plasticity highlight the brain’s remarkable capacity to adapt under sensory deprivation, with deprived regions being captured to process other sensory modalities. Furthermore, the observation of similar plasticity processes across species raises questions about the evolutionary conservation of these mechanisms and how they have been shaped by evolutionary pressures across mammals. Such findings provide key insights into neural plasticity and have significant implications for understanding and leveraging these mechanisms in sensory rehabilitation.

## Future perspectives

The findings suggest that intrinsic mechanisms within the thalamus drive the early segregation of nuclei, even before thalamocortical connectivity is fully established. Similar to processes observed in the cortex^231^ it is likely that neuronal progenitors transmit distinct transcriptomic and epigenetic profiles to their progeny, laying the foundation for the specification and functional organization of thalamic nuclei.

The advent of scRNA-seq has revolutionized the study of thalamic development by providing a comprehensive, high-resolution view of gene expression. This approach enables the profiling of transcriptional diversity at the single-cell level, revealing cellular heterogeneity within sensory-specific nuclei that would otherwise remain obscured in bulk analyses. However, despite these advances, transcription factors and their regulatory networks are still poorly characterized, partly due to the inability of scRNA-seq to directly capture regulatory elements or chromatin accessibility.

To address these limitations, techniques such as single-cell sequencing assay for transposase-accessible chromatin (scATAC-seq) and multiome sequencing, complemented by lineage-tracing barcoding, have emerged as promising tools. scATAC-seq allows for the identification of active regulatory regions and transcription factor binding sites critical to thalamic development. When combined with single-cell transcriptomics in a multiomic framework, these techniques provide a powerful means to integrate gene expression profiles with regulatory landscapes, elucidating how specific transcription factors drive sensory modality-specific differentiation. Such tools could unravel the intricate transcriptional networks underlying thalamic differentiation and advance our understanding of how sensory modalities are specified^232,233^. This transfer of molecular identity likely represents a crucial initial step in the hierarchical development of the thalamus. Nonetheless, further research is needed to validate this hypothesis and to investigate how these intrinsic mechanisms interact with external cues, such as peripheral sensory input, to refine and stabilize thalamic circuit organization.

While high-throughput molecular techniques offer insights into gene regulation and cell-type diversity, complementary physiological and computational methods are essential to fully understand thalamocortical development. *In vivo* calcium imaging and multi-electrode array recordings reveal functional dynamics of thalamic waves and their role in shaping connectivity. Recent studies suggest that these spontaneous activity patterns contribute to sensory map refinement^39,45,194^, though their cellular mechanisms are not fully understood. Computational models can simulate interactions between molecular gradients, activity, and axonal pathfinding, offering testable hypotheses about how intrinsic transcriptional programs and external inputs coordinate thalamic circuit assembly. Integrating high-throughput molecular data with electrophysiological insights remains challenging, but technologies like Patch-seq and single cell transcriptomics are key to linking functional properties to molecular identity, enhancing our understanding of thalamic circuit assembly^234^. Additionally, spatial transcriptomics approaches^235^ enable the mapping of gene expression patterns within intact tissues, offering further insights into the spatial organization of thalamocortical circuits. As recently discussed by Dooley and van der Heijden^236^ such integrative approaches are still underutilized in developmental neuroscience but are critical for bridging molecular and functional perspectives on thalamocortical development.

Although much progress has been done on determining the molecular mechanisms that control TCA pathfinding through the ventral telencephalon and into the cortex, a similar understanding of the mechanisms controlling area-specific thalamocortical targeting is still lacking. Cell adhesion molecules may mediate this process, as is the case for the retinotectal projection^237^. For instance, thalamic sensory nuclei and their target primary sensory cortices show matching patterns of expression of cadherins^238–240^. Vanderhaegen and colleagues showed that ephrin-A5 is expressed in a medial to lateral gradient in S1, while its receptor, EphA4, is matched by the same gradient in the VPM of the thalamus, from which it receives direct input^71^. Lhx2, a LIM-homeodomain transcription factor, is highly expressed in the primary auditory area^129^ and the MGv that projects to it^241^. Although these are intriguing results, suggestive of a ligand-receptor molecular code that guides synaptic partner matching, confirmation of such a mechanism is still lacking. The availability of spatial transcriptomics and bioinformatic tools to explore cell-cell communication^242^ or recent advances in growth cone purification and sequencing^243^ may provide new insights about the molecular mechanisms underlying TCA specific targeting.

## Box 1 The thalamocortical loop

Thalamic FO nuclei, including the dorsal lateral geniculate nucleus (dLG) for vision, the ventral posterior medial nucleus (VPM) for somatosensation, and the ventral medial geniculate nucleus (MGv) for audition, receive inputs from peripheral sensory organs and send their axons to primary sensory cortical areas, the primary visual cortex (V1), primary somatosensory cortex (S1) and the primary auditory cortex (A1), respectively. These axons primarily target layer 4 of their respective cortical regions but also innervate other cortical layers. Thalamic HO nuclei, including the latero-posterior (LP) nucleus for vision, the posteromedial (POm) nucleus for somatosensation and the dorsomedial geniculate (MGd) nucleus for audition, receive their primary input from cortical regions and form part of the cortico-thalamo-cortical pathways. This transthalamic route provides a feedforward and bidirectional communication between the cortex and thalamus, facilitating the processing and integration of sensory information at multiple levels. In sum, for each sensory modality, the primary sensory cortex receives input from the exteroceptive FO nucleus, and projects back to the corresponding HO thalamic nucleus, which then targets a secondary sensory cortex^244^.

## References

[1] Halassa MM (2022). The Thalamus.

[2] Rikhye RV, Gilra A, Halassa MM (2018). Thalamic regulation of switching between cortical representations enables cognitive flexibility. Nat Neurosci.

[3] Vertes RP, Linley SB, Groenewegen HJ, Witter MP, Paxinos George (2015). The Rat Nervous System.

[4] Gezelius H, Lopez-Bendito G (2017). Thalamic neuronal specification and early circuit formation. Dev Neurobiol.

[5] Jones EG (2007). The Thalamus.

[6] Harris KD, Shepherd GM (2015). The neocortical circuit: themes and variations. Nat Neurosci.

[7] Theyel BB, Llano DA, Sherman SM (2010). The corticothalamocortical circuit drives higher-order cortex in the mouse. Nat Neurosci.

[8] Sherman SM, Guillery RW (2011). Distinct functions for direct and transthalamic corticocortical connections. J Neurophysiol.

[9] Clascá F, Rubio-Garrido P, Jabaudon D (2012). Unveiling the diversity of thalamocortical neuron subtypes. Eur J Neurosci.

[10] Landisman CE, Connors BW (2007). VPM and PoM nuclei of the rat somatosensory thalamus: intrinsic neuronal properties and corticothalamic feedback. Cereb Cortex.

[11] Li J, Bickford ME, Guido W (2003). Distinct firing properties of higher order thalamic relay neurons. J Neurophysiol.

[12] Vue TY (2007). Characterization of progenitor domains in the developing mouse thalamus. J Comp Neurol.

[13] Phillips JW (2019). A repeated molecular architecture across thalamic pathways. Nat Neurosci.

[14] Nagalski A (2016). Molecular anatomy of the thalamic complex and the underlying transcription factors. Brain Struct Funct.

[15] Frangeul L (2016). A cross-modal genetic framework for the development and plasticity of sensory pathways. Nature.

[16] Govek KW (2022). Developmental trajectories of thalamic progenitors revealed by single-cell transcriptome profiling and Shh perturbation. Cell Rep.

[17] Lo Giudice Q, Wagener RJ, Abe P, Frangeul L, Jabaudon D (2024). Developmental emergence of first- and higher-order thalamic neuron molecular identities. Development.

[18] Gezelius H (2017). Genetic Labeling of Nuclei-Specific Thalamocortical Neurons Reveals Putative Sensory-Modality Specific Genes. Cereb Cortex.

[19] Bulfone A (1993). Spatially restricted expression of Dlx-1, Dlx-2 (Tes-1), Gbx-2, and Wnt-3 in the embryonic day 12.5 mouse forebrain defines potential transverse and longitudinal segmental boundaries. J Neurosci.

[20] Echevarría D, Vieira C, Gimeno L, Martínez S (2003). Neuroepithelial secondary organizers and cell fate specification in the developing brain. Brain Res Brain Res Rev.

[21] Puelles L (2024). Functional Implications of the Prosomeric Brain Model. Biomolecules.

[22] Puelles L, Harrison M, Paxinos G, Watson C (2013). A developmental ontology for the mammalian brain based on the prosomeric model. Trends Neurosci.

[23] Puelles L, Rubenstein JL (2003). Forebrain gene expression domains and the evolving prosomeric model. Trends Neurosci.

[24] Hagemann AI, Scholpp S (2012). The Tale of the Three Brothers - Shh, Wnt, and Fgf during Development of the Thalamus. Front Neurosci.

[25] Vue TY (2009). Sonic hedgehog signaling controls thalamic progenitor identity and nuclei specification in mice. J Neurosci.

[26] Fode C (2000). A role for neural determination genes in specifying the dorsoventral identity of telencephalic neurons. Genes Dev.

[27] Hippenmeyer S (2010). Genetic mosaic dissection of Lis1 and Ndel1 in neuronal migration. Neuron.

[28] Shi W (2017). Ontogenetic establishment of order-specific nuclear organization in the mammalian thalamus. Nat Neurosci.

[29] Wong SZH (2018). In vivo clonal analysis reveals spatiotemporal regulation of thalamic nucleogenesis. PLoS Biol.

[30] Angevine JB (1970). Time of neuron origin in the diencephalon of the mouse. An autoradiographic study. J Comp Neurol.

[31] Guo Q, Li JYH (2019). Defining developmental diversification of diencephalon neurons through single cell gene expression profiling. Development.

[32] Wang L, Bluske KK, Dickel LK, Nakagawa Y (2011). Basal progenitor cells in the embryonic mouse thalamus - their molecular characterization and the role of neurogenins and Pax6. Neural Dev.

[33] Thor S (2024). Indirect neurogenesis in space and time. Nat Rev Neurosci.

[34] Golding B (2014). Retinal Input Directs the Recruitment of Inhibitory Interneurons into Thalamic Visual Circuits. Neuron.

[35] Herrero-Navarro Á (2021). Astrocytes and neurons share region-specific transcriptional signatures that confer regional identity to neuronal reprogramming. Sci Adv.

[36] Akdemir ES, Huang AY, Deneen B (2020). Astrocytogenesis: where, when, and how. F1000Res.

[37] Moreno-Juan V (2017). Prenatal thalamic waves regulate cortical area size prior to sensory processing. Nat Commun.

[38] Martini FJ, Moreno-Juan V, Filipchuk A, Valdeolmillos M, Lopez-Bendito G (2018). Impact of thalamocortical input on barrel cortex development. Neuroscience.

[39] Martini FJ, Guillamon-Vivancos T, Moreno-Juan V, Valdeolmillos M, Lopez-Bendito G (2021). Spontaneous Activity in Developing Thalamic and Cortical Sensory Networks. Neuron.

[40] Herrmann K, Shatz CJ (1995). Blockade of action potential activity alters initial arborization of thalamic axons within cortical layer 4. Proc Natl Acad Sci U S A.

[41] Uesaka N, Hayano Y, Yamada A, Yamamoto N (2007). Interplay between laminar specificity and activity-dependent mechanisms of thalamocortical axon branching. J Neurosci.

[42] Mire E (2012). Spontaneous activity regulates Robo1 transcription to mediate a switch in thalamocortical axon growth. Nat Neurosci.

[43] Yamamoto N, López-Bendito G (2012). Shaping brain connections through spontaneous neural activity. Eur J Neurosci.

[44] Castillo-Paterna M (2015). DCC functions as an accelerator of thalamocortical axonal growth downstream of spontaneous thalamic activity. EMBO Rep.

[45] Antón-Bolaños N (2019). Prenatal activity from thalamic neurons governs the emergence of functional cortical maps in mice. Science.

[46] Cang J, Feldheim DA (2013). Developmental mechanisms of topographic map formation and alignment. Annu Rev Neurosci.

[47] Faust TE, Gunner G, Schafer DP (2021). Mechanisms governing activity-dependent synaptic pruning in the developing mammalian CNS. Nat Rev Neurosci.

[48] Fitzgibbon T (2007). Do first order and higher order regions of the thalamic reticular nucleus have different developmental timetables?. Exp Neurol.

[49] Hanganu-Opatz IL (2010). Between molecules and experience: role of early patterns of coordinated activity for the development of cortical maps and sensory abilities. Brain Res Rev.

[50] Molnar Z, Luhmann HJ, Kanold PO (2020). Transient cortical circuits match spontaneous and sensory-driven activity during development. Science.

[51] O’Donnell P, Grace AA (1998). Dysfunctions in multiple interrelated systems as the neurobiological bases of schizophrenic symptom clusters. Schizophr Bull.

[52] Herrera CG, Tarokh L (2024). A Thalamocortical Perspective on Sleep Spindle Alterations in Neurodevelopmental Disorders. Curr Sleep Med Rep.

[53] Benoit LJ, Canetta S, Kellendonk C (2022). Thalamocortical Development: A Neurodevelopmental Framework for Schizophrenia. Biol Psychiatry.

[54] López-Bendito G, Molnár Z (2003). Thalamocortical development: how are we going to get there?. Nat Rev Neurosci.

[55] Khazipov R, Minlebaev M, Valeeva G (2013). Early gamma oscillations. Neuroscience.

[56] Murata Y, Colonnese MT (2016). An excitatory cortical feedback loop gates retinal wave transmission in rodent thalamus. Elife.

[57] Minlebaev M, Colonnese M, Tsintsadze T, Sirota A, Khazipov R (2011). Early γ oscillations synchronize developing thalamus and cortex. Science.

[58] Yang JW (2013). Thalamic network oscillations synchronize ontogenetic columns in the newborn rat barrel cortex. Cereb Cortex.

[59] Murata Y, Colonnese MT (2018). Thalamus Controls Development and Expression of Arousal States in Visual Cortex. J Neurosci.

[60] Golshani P (2009). Internally mediated developmental desynchronization of neocortical network activity. J Neurosci.

[61] Mizuno H (2018). Patchwork-Type Spontaneous Activity in Neonatal Barrel Cortex Layer 4 Transmitted via Thalamocortical Projections. Cell Rep.

[62] Madisen L (2015). Transgenic mice for intersectional targeting of neural sensors and effectors with high specificity and performance. Neuron.

[63] Gribizis A (2019). Visual Cortex Gains Independence from Peripheral Drive before Eye Opening. Neuron.

[64] Chatterjee M (2012). Gbx2 regulates thalamocortical axon guidance by modifying the LIM and Robo codes. Development.

[65] Marcos-Mondéjar P (2012). The lhx2 transcription factor controls thalamocortical axonal guidance by specific regulation of robo1 and robo2 receptors. J Neurosci.

[66] Mallika C, Guo Q, Li JY (2015). Gbx2 is essential for maintaining thalamic neuron identity and repressing habenular characters in the developing thalamus. Dev Biol.

[67] Ebisu H, Iwai-Takekoshi L, Fujita-Jimbo E, Momoi T, Kawasaki H (2017). Foxp2 Regulates Identities and Projection Patterns of Thalamic Nuclei During Development. Cereb Cortex.

[68] Gao PP (1998). Regulation of thalamic neurite outgrowth by the Eph ligand ephrin-A5: implications in the development of thalamocortical projections. Proc Natl Acad Sci U S A.

[69] Mackarehtschian K, Lau CK, Caras I, McConnell SK (1999). Regional differences in the developing cerebral cortex revealed by ephrin-A5 expression. Cereb Cortex.

[70] Uziel D (2002). Miswiring of limbic thalamocortical projections in the absence of ephrin-A5. J Neurosci.

[71] Vanderhaeghen P (2000). A mapping label required for normal scale of body representation in the cortex. Nat Neurosci.

[72] Takemoto M (2002). Ephrin-B3-EphA4 interactions regulate the growth of specific thalamocortical axon populations in vitro. Eur J Neurosci.

[73] Dufour A (2003). Area specificity and topography of thalamocortical projections are controlled by ephrin/Eph genes. Neuron.

[74] Huang EJ, Reichardt LF (2001). Neurotrophins: roles in neuronal development and function. Annu Rev Neurosci.

[75] Cabelli RJ, Shelton DL, Segal RA, Shatz CJ (1997). Blockade of endogenous ligands of trkB inhibits formation of ocular dominance columns. Neuron.

[76] Xu B (2000). Cortical degeneration in the absence of neurotrophin signaling: dendritic retraction and neuronal loss after removal of the receptor TrkB. Neuron.

[77] Jiao Y (2011). A key mechanism underlying sensory experience-dependent maturation of neocortical GABAergic circuits in vivo. Proc Natl Acad Sci U S A.

[78] Lu B, Nagappan G, Lu Y (2014). BDNF and synaptic plasticity, cognitive function, and dysfunction. Handb Exp Pharmacol.

[79] Park H, Poo MM (2013). Neurotrophin regulation of neural circuit development and function. Nat Rev Neurosci.

[80] Bortolotto ZA, Fitzjohn SM, Collingridge GL (1999). Roles of metabotropic glutamate receptors in LTP and LTD in the hippocampus. Curr Opin Neurobiol.

[81] Hannan AJ (2001). PLC-beta1, activated via mGluRs, mediates activity-dependent differentiation in cerebral cortex. Nat Neurosci.

[82] Cases O (1996). Lack of barrels in the somatosensory cortex of monoamine oxidase A-deficient mice: role of a serotonin excess during the critical period. Neuron.

[83] Salichon N (2001). Excessive activation of serotonin (5-HT) 1B receptors disrupts the formation of sensory maps in monoamine oxidase a and 5-ht transporter knock-out mice. J Neurosci.

[84] Matsumoto N, Hoshiko M, Sugo N, Fukazawa Y, Yamamoto N (2016). Synapse-dependent and independent mechanisms of thalamocortical axon branching are regulated by neuronal activity. Dev Neurobiol.

[85] Kim JI (2024). Human assembloids reveal the consequences of CACNA1G gene variants in the thalamocortical pathway. Neuron.

[86] Golshani P, Hutnick L, Schweizer F, Fan G (2005). Conditional Dnmt1 deletion in dorsal forebrain disrupts development of somatosensory barrel cortex and thalamocortical long-term potentiation. Thalamus Relat Syst.

[87] Pumo GM, Kitazawa T, Rijli FM (2022). Epigenetic and Transcriptional Regulation of Spontaneous and Sensory Activity Dependent Programs During Neuronal Circuit Development. Front Neural Circuits.

[88] Fetter-Pruneda I (2013). Shifts in developmental timing, and not increased levels of experience-dependent neuronal activity, promote barrel expansion in the primary somatosensory cortex of rats enucleated at birth. PLoS One.

[89] Alchini R (2017). Nucleocytoplasmic Shuttling of Histone Deacetylase 9 Controls Activity-Dependent Thalamocortical Axon Branching. Sci Rep.

[90] Cadwell CR, Bhaduri A, Mostajo-Radji MA, Keefe MG, Nowakowski TJ (2019). Development and Arealization of the Cerebral Cortex. Neuron.

[91] Mitrofanis J, Guillery RW (1993). New views of the thalamic reticular nucleus in the adult and the developing brain. Trends Neurosci.

[92] Métin C, Godement P (1996). The ganglionic eminence may be an intermediate target for corticofugal and thalamocortical axons. J Neurosci.

[93] Molnár Z, Adams R, Blakemore C (1998). Mechanisms underlying the early establishment of thalamocortical connections in the rat. J Neurosci.

[94] Braisted JE, Tuttle R, O’leary DD (1999). Thalamocortical axons are influenced by chemorepellent and chemoattractant activities localized to decision points along their path. Dev Biol.

[95] Braisted JE (2000). Netrin-1 promotes thalamic axon growth and is required for proper development of the thalamocortical projection. J Neurosci.

[96] Tuttle R, Nakagawa Y, Johnson JE, O’Leary DD (1999). Defects in thalamocortical axon pathfinding correlate with altered cell domains in Mash-1-deficient mice. Development.

[97] Garel S, Marín F, Grosschedl R, Charnay P (1999). Ebf1 controls early cell differentiation in the embryonic striatum. Development.

[98] Garel S, Yun K, Grosschedl R, Rubenstein JL (2002). The early topography of thalamocortical projections is shifted in Ebf1 and Dlx1/2 mutant mice. Development.

[99] Marín O, Baker J, Puelles L, Rubenstein JL (2002). Patterning of the basal telencephalon and hypothalamus is essential for guidance of cortical projections. Development.

[100] López-Bendito G (2006). Tangential neuronal migration controls axon guidance: a role for neuregulin-1 in thalamocortical axon navigation. Cell.

[101] Edwards MA, Yamamoto M, Caviness VS (1990). Organization of radial glia and related cells in the developing murine CNS. An analysis based upon a new monoclonal antibody marker. Neuroscience.

[102] Stoykova A, Götz M, Gruss P, Price J (1997). Pax6-dependent regulation of adhesive patterning, R-cadherin expression and boundary formation in developing forebrain. Development.

[103] Hartfuss E, Galli R, Heins N, Götz M (2001). Characterization of CNS precursor subtypes and radial glia. Dev Biol.

[104] Carney RS (2006). Cell migration along the lateral cortical stream to the developing basal telencephalic limbic system. J Neurosci.

[105] Molnár Z, Cordery P (1999). Connections between cells of the internal capsule, thalamus, and cerebral cortex in embryonic rat. J Comp Neurol.

[106] Chen Y, Magnani D, Theil T, Pratt T, Price DJ (2012). Evidence that descending cortical axons are essential for thalamocortical axons to cross the pallial-subpallial boundary in the embryonic forebrain. PLoS One.

[107] Molnár Z, Blakemore C (1995). How do thalamic axons find their way to the cortex?. Trends Neurosci.

[108] Hevner RF, Miyashita-Lin E, Rubenstein JL (2002). Cortical and thalamic axon pathfinding defects in Tbr1, Gbx2, and Pax6 mutant mice: evidence that cortical and thalamic axons interact and guide each other. J Comp Neurol.

[109] Jones L, López-Bendito G, Gruss P, Stoykova A, Molnár Z (2002). Pax6 is required for the normal development of the forebrain axonal connections. Development.

[110] López-Bendito G, Chan CH, Mallamaci A, Parnavelas J, Molnár Z (2002). Role of Emx2 in the development of the reciprocal connectivity between cortex and thalamus. J Comp Neurol.

[111] Kanold PO, Luhmann HJ (2010). The subplate and early cortical circuits. Annu Rev Neurosci.

[112] Price DJ, Aslam S, Tasker L, Gillies K (1997). Fates of the earliest generated cells in the developing murine neocortex. J Comp Neurol.

[113] Ghosh A, Antonini A, McConnell SK, Shatz CJ (1990). Requirement for subplate neurons in the formation of thalamocortical connections. Nature.

[114] Ghosh A, Shatz CJ (1992). Involvement of subplate neurons in the formation of ocular dominance columns. Science.

[115] Kanold PO, Kara P, Reid RC, Shatz CJ (2003). Role of subplate neurons in functional maturation of visual cortical columns. Science.

[116] Ghosh A, Shatz CJ (1992). Pathfinding and target selection by developing geniculocortical axons. J Neurosci.

[117] Naegele JR, Jhaveri S, Schneider GE (1988). Sharpening of topographical projections and maturation of geniculocortical axon arbors in the hamster. J Comp Neurol.

[118] Hanganu IL, Kilb W, Luhmann HJ (2002). Functional synaptic projections onto subplate neurons in neonatal rat somatosensory cortex. J Neurosci.

[119] Herrmann K, Antonini A, Shatz CJ (1994). Ultrastructural evidence for synaptic interactions between thalamocortical axons and subplate neurons. Eur J Neurosci.

[120] Higashi S, Molnár Z, Kurotani T, Toyama K (2002). Prenatal development of neural excitation in rat thalamocortical projections studied by optical recording. Neuroscience.

[121] Hirsch S, Luhmann HJ (2008). Pathway-specificity in N-methyl-D-aspartate receptor-mediated synaptic inputs onto subplate neurons. Neuroscience.

[122] Catalano SM, Shatz CJ (1998). Activity-dependent cortical target selection by thalamic axons. Science.

[123] Doyle DZ (2021). Chromatin remodeler. Proc Natl Acad Sci U S A.

[124] Yamamoto N, Higashi S, Toyama K (1997). Stop and branch behaviors of geniculocortical axons: a time-lapse study in organotypic cocultures. J Neurosci.

[125] Yamamoto N (2000). Characterization of factors regulating lamina-specific growth of thalamocortical axons. J Neurobiol.

[126] Yamamoto N (2002). Cellular and molecular basis for the formation of lamina-specific thalamocortical projections. Neurosci Res.

[127] Donoghue MJ, Rakic P (1999). Molecular evidence for the early specification of presumptive functional domains in the embryonic primate cerebral cortex. J Neurosci.

[128] Donoghue MJ, Rakic P (1999). Molecular gradients and compartments in the embryonic primate cerebral cortex. Cereb Cortex.

[129] Nakagawa Y, Johnson JE, O’Leary DD (1999). Graded and areal expression patterns of regulatory genes and cadherins in embryonic neocortex independent of thalamocortical input. J Neurosci.

[130] Miyashita-Lin EM, Hevner R, Wassarman KM, Martinez S, Rubenstein JL (1999). Early neocortical regionalization in the absence of thalamic innervation. Science.

[131] Gulisano M, Broccoli V, Pardini C, Boncinelli E (1996). Emx1 and Emx2 show different patterns of expression during proliferation and differentiation of the developing cerebral cortex in the mouse. Eur J Neurosci.

[132] Stoykova A, Gruss P (1994). Roles of Pax-genes in developing and adult brain as suggested by expression patterns. J Neurosci.

[133] Bishop KM, Goudreau G, O’Leary DD (2000). Regulation of area identity in the mammalian neocortex by Emx2 and Pax6. Science.

[134] Mallamaci A, Muzio L, Chan CH, Parnavelas J, Boncinelli E (2000). Area identity shifts in the early cerebral cortex of Emx2-/- mutant mice. Nat Neurosci.

[135] Clegg JM (2015). Pax6 is required intrinsically by thalamic progenitors for the normal molecular patterning of thalamic neurons but not the growth and guidance of their axons. Neural Dev.

[136] Georgala PA, Carr CB, Price DJ (2011). The role of Pax6 in forebrain development. Dev Neurobiol.

[137] Quintana-Urzainqui I (2020). The role of the diencephalon in the guidance of thalamocortical axons in mice. Development.

[138] Liu Q, Dwyer ND, O’Leary DD (2000). Differential expression of COUP-TFI, CHL1, and two novel genes in developing neocortex identified by differential display PCR. J Neurosci.

[139] Zhou C, Tsai SY, Tsai MJ (2001). COUP-TFI: an intrinsic factor for early regionalization of the neocortex. Genes Dev.

[140] Qiu Y (1994). Spatiotemporal expression patterns of chicken ovalbumin upstream promoter-transcription factors in the developing mouse central nervous system: evidence for a role in segmental patterning of the diencephalon. Proc Natl Acad Sci U S A.

[141] Crossley PH, Martin GR (1995). The mouse Fgf8 gene encodes a family of polypeptides and is expressed in regions that direct outgrowth and patterning in the developing embryo. Development.

[142] Crossley PH, Martinez S, Ohkubo Y, Rubenstein JL (2001). Coordinate expression of Fgf8, Otx2, Bmp4, and Shh in the rostral prosencephalon during development of the telencephalic and optic vesicles. Neuroscience.

[143] Grove EA, Tole S, Limon J, Yip L, Ragsdale CW (1998). The hem of the embryonic cerebral cortex is defined by the expression of multiple Wnt genes and is compromised in Gli3-deficient mice. Development.

[144] Furuta Y, Piston DW, Hogan BL (1997). Bone morphogenetic proteins (BMPs) as regulators of dorsal forebrain development. Development.

[145] Shimamura K, Hartigan DJ, Martinez S, Puelles L, Rubenstein JL (1995). Longitudinal organization of the anterior neural plate and neural tube. Development.

[146] Callejas-Marin A (2022). Mediated. Front Neuroanat.

[147] Fukuchi-Shimogori T, Grove EA (2001). Neocortex patterning by the secreted signaling molecule FGF8. Science.

[148] Shimogori T, Grove EA (2005). Fibroblast growth factor 8 regulates neocortical guidance of area-specific thalamic innervation. J Neurosci.

[149] Abe P (2015). Intermediate Progenitors Facilitate Intracortical Progression of Thalamocortical Axons and Interneurons through CXCL12 Chemokine Signaling. J Neurosci.

[150] Wagener RJ (2016). Thalamocortical Connections Drive Intracortical Activation of Functional Columns in the Mislaminated Reeler Somatosensory Cortex. Cereb Cortex.

[151] Guillamón-Vivancos T (2022). Input-dependent segregation of visual and somatosensory circuits in the mouse superior colliculus. Science.

[152] Dupont E, Hanganu IL, Kilb W, Hirsch S, Luhmann HJ (2006). Rapid developmental switch in the mechanisms driving early cortical columnar networks. Nature.

[153] Singh MB, White JA, McKimm EJ, Milosevic MM, Antic SD (2019). Mechanisms of Spontaneous Electrical Activity in the Developing Cerebral Cortex-Mouse Subplate Zone. Cereb Cortex.

[154] Luhmann HJ, Kilb W, Hanganu-Opatz IL (2009). Subplate cells: amplifiers of neuronal activity in the developing cerebral cortex. Front Neuroanat.

[155] Tolner EA, Sheikh A, Yukin AY, Kaila K, Kanold PO (2012). Subplate neurons promote spindle bursts and thalamocortical patterning in the neonatal rat somatosensory cortex. J Neurosci.

[156] Kanold PO, Shatz CJ (2006). Subplate neurons regulate maturation of cortical inhibition and outcome of ocular dominance plasticity. Neuron.

[157] Tuncdemir SN (2016). Early Somatostatin Interneuron Connectivity Mediates the Maturation of Deep Layer Cortical Circuits. Neuron.

[158] Marques-Smith A (2016). A Transient Translaminar GABAergic Interneuron Circuit Connects Thalamocortical Recipient Layers in Neonatal Somatosensory Cortex. Neuron.

[159] Dwivedi D (2024). Metabotropic signaling within somatostatin interneurons controls transient thalamocortical inputs during development. Nat Commun.

[160] Mizuno H (2014). NMDAR-regulated dynamics of layer 4 neuronal dendrites during thalamocortical reorganization in neonates. Neuron.

[161] De León Reyes NS (2019). Transient callosal projections of L4 neurons are eliminated for the acquisition of local connectivity. Nat Commun.

[162] Ibrahim LA (2021). Bottom-up inputs are required for establishment of top-down connectivity onto cortical layer 1 neurogliaform cells. Neuron.

[163] Chou SJ (2013). Geniculocortical input drives genetic distinctions between primary and higher-order visual areas. Science.

[164] Vue TY (2013). Thalamic control of neocortical area formation in mice. J Neurosci.

[165] Pouchelon G (2014). Modality-specific thalamocortical inputs instruct the identity of postsynaptic L4 neurons. Nature.

[166] Li H (2013). Laminar and columnar development of barrel cortex relies on thalamocortical neurotransmission. Neuron.

[167] Matsui A (2013). BTBD3 controls dendrite orientation toward active axons in mammalian neocortex. Science.

[168] Young TR (2023). Thalamocortical control of cell-type specificity drives circuits for processing whisker-related information in mouse barrel cortex. Nat Commun.

[169] Guillamon-Vivancos T (2019). Distinct Neocortical Progenitor Lineages Fine-tune Neuronal Diversity in a Layer-specific Manner. Cereb Cortex.

[170] Buchan MJ (2024). Higher-order thalamocortical circuits are specified by embryonic cortical progenitor types in the mouse brain. Cell Rep.

[171] Blumberg MS, Coleman CM, Gerth AI, McMurray B (2013). Spatiotemporal structure of REM sleep twitching reveals developmental origins of motor synergies. Curr Biol.

[172] Inácio AR, Nasretdinov A, Lebedeva J, Khazipov R (2016). Sensory feedback synchronizes motor and sensory neuronal networks in the neonatal rat spinal cord. Nat Commun.

[173] Kennedy HJ (2012). New developments in understanding the mechanisms and function of spontaneous electrical activity in the developing mammalian auditory system. J Assoc Res Otolaryngol.

[174] Leighton AH, Lohmann C (2016). The Wiring of Developing Sensory Circuits-From Patterned Spontaneous Activity to Synaptic Plasticity Mechanisms. Front Neural Circuits.

[175] Wang HC, Bergles DE (2015). Spontaneous activity in the developing auditory system. Cell Tissue Res.

[176] Geal-Dor M, Freeman S, Li G, Sohmer H (1993). Development of hearing in neonatal rats: air and bone conducted ABR thresholds. Hear Res.

[177] Blankenship AG, Feller MB (2010). Mechanisms underlying spontaneous patterned activity in developing neural circuits. Nat Rev Neurosci.

[178] López-Bendito G, Aníbal-Martínez M, Martini FJ (2022). Cross-Modal Plasticity in Brains Deprived of Visual Input Before Vision. Annu Rev Neurosci.

[179] Cang J (2005). Development of precise maps in visual cortex requires patterned spontaneous activity in the retina. Neuron.

[180] Burbridge TJ (2014). Visual circuit development requires patterned activity mediated by retinal acetylcholine receptors. Neuron.

[181] Dooley JC, Krubitzer LA (2019). Alterations in cortical and thalamic connections of somatosensory cortex following early loss of vision. J Comp Neurol.

[182] Dye CA, Abbott CW, Huffman KJ (2012). Bilateral enucleation alters gene expression and intraneocortical connections in the mouse. Neural Dev.

[183] Izraeli R (2002). Cross-modal neuroplasticity in neonatally enucleated hamsters: structure, electrophysiology and behaviour. Eur J Neurosci.

[184] Karlen SJ, Krubitzer L (2009). Effects of bilateral enucleation on the size of visual and nonvisual areas of the brain. Cereb Cortex.

[185] Rhoades RW, Mooney RD, Fish SE (1984). A comparison of visual callosal organization in normal, bilaterally enucleated and congenitally anophthalmic mice. Exp Brain Res.

[186] Williams AL, Reese BE, Jeffery G (2002). Role of retinal afferents in regulating growth and shape of the lateral geniculate nucleus. J Comp Neurol.

[187] Dehay C, Giroud P, Berland M, Killackey H, Kennedy H (1996). Contribution of thalamic input to the specification of cytoarchitectonic cortical fields in the primate: effects of bilateral enucleation in the fetal monkey on the boundaries, dimensions, and gyrification of striate and extrastriate cortex. J Comp Neurol.

[188] Sur M, Garraghty PE, Roe AW (1988). Experimentally induced visual projections into auditory thalamus and cortex. Science.

[189] Hensch TK (2005). Critical period mechanisms in developing visual cortex. Curr Top Dev Biol.

[190] Hooks BM, Chen C (2007). Critical periods in the visual system: changing views for a model of experience-dependent plasticity. Neuron.

[191] Reh RK (2020). Critical period regulation across multiple timescales. Proc Natl Acad Sci U S A.

[192] Barkat TR, Polley DB, Hensch TK (2011). A critical period for auditory thalamocortical connectivity. Nat Neurosci.

[193] Erzurumlu RS, Gaspar P (2012). Development and critical period plasticity of the barrel cortex. Eur J Neurosci.

[194] Aníbal-Martínez M (2025). A prenatal window for enhancing spatial resolution of cortical barrel maps. Nat Commun.

[195] Sadato N (1996). Activation of the primary visual cortex by Braille reading in blind subjects. Nature.

[196] Woolsey TA, Van der Loos H (1970). The structural organization of layer IV in the somatosensory region (SI) of mouse cerebral cortex. The description of a cortical field composed of discrete cytoarchitectonic units. Brain Res.

[197] Belford GR, Killackey HP (1979). The development of vibrissae representation in subcortical trigeminal centers of the neonatal rat. J Comp Neurol.

[198] Killackey HP, Dawson DR (1989). Expansion of the Central Hindpaw Representation Following Fetal Forelimb Removal in the Rat. Eur J Neurosci.

[199] Woolsey TA, Anderson JR, Wann JR, Stanfield BB (1979). Effects of early vibrissae damage on neurons in the ventrobasal (VB) thalamus of the mouse. J Comp Neurol.

[200] Killackey HP, Belford G, Ryugo R, Ryugo DK (1976). Anomalous organization of thalamocortical projections consequent to vibrissae removal in the newborn rat and mouse. Brain Res.

[201] Renier N (2017). A mutant with bilateral whisker to barrel inputs unveils somatosensory mapping rules in the cerebral cortex. Elife.

[202] Woolsey TA, Wann JR (1976). Areal changes in mouse cortical barrels following vibrissal damage at different postnatal ages. J Comp Neurol.

[203] Hubel DH, Wiesel TN (1963). Receptive Fields of Cells in Striate Cortex of Very Young, Visually Inexperienced Kittens. J Neurophysiol.

[204] Craddock R, Vasalauskaite A, Ranson A, Sengpiel F (2023). Experience dependent plasticity of higher visual cortical areas in the mouse. Cereb Cortex.

[205] Takahata T (2024). Development of ocular dominance columns across rodents and other species: revisiting the concept of critical period plasticity. Front Neural Circuits.

[206] Allen CB, Celikel T, Feldman DE (2003). Long-term depression induced by sensory deprivation during cortical map plasticity in vivo. Nat Neurosci.

[207] Feldman DE, Brecht M (2005). Map plasticity in somatosensory cortex. Science.

[208] Petersen CC (2007). The functional organization of the barrel cortex. Neuron.

[209] Foeller E, Feldman DE (2004). Synaptic basis for developmental plasticity in somatosensory cortex. Curr Opin Neurobiol.

[210] Goldreich D, Kanics IM (2006). Performance of blind and sighted humans on a tactile grating detection task. Percept Psychophys.

[211] Renier L (2013). Right occipital cortex activation correlates with superior odor processing performance in the early blind. PLoS One.

[212] Röder B (1999). Improved auditory spatial tuning in blind humans. Nature.

[213] Bavelier D, Neville HJ (2002). Cross-modal plasticity: where and how?. Nat Rev Neurosci.

[214] Lomber SG, Meredith MA, Kral A (2010). Cross-modal plasticity in specific auditory cortices underlies visual compensations in the deaf. Nat Neurosci.

[215] Zimmermann M, Cusack R, Bedny M, Szwed M (2024). Auditory areas are recruited for naturalistic visual meaning in early deaf people. Nat Commun.

[216] Auer ET, Bernstein LE, Sungkarat W, Singh M (2007). Vibrotactile activation of the auditory cortices in deaf versus hearing adults. Neuroreport.

[217] Cardon G, Sharma A (2018). Somatosensory Cross-Modal Reorganization in Adults With Age-Related, Early-Stage Hearing Loss. Front Hum Neurosci.

[218] Kozanian OO, Abbott CW, Huffman KJ (2015). Rapid Changes in Cortical and Subcortical Brain Regions after Early Bilateral Enucleation in the Mouse. PLoS One.

[219] Mezzera C, López-Bendito G (2016). Cross-modal plasticity in sensory deprived animal models: From the thalamocortical development point of view. J Chem Neuroanat.

[220] Chabot N (2007). Audition differently activates the visual system in neonatally enucleated mice compared with anophthalmic mutants. Eur J Neurosci.

[221] Rauschecker JP, Korte M (1993). Auditory compensation for early blindness in cat cerebral cortex. J Neurosci.

[222] Abbott CW, Kozanian OO, Huffman KJ (2015). The effects of lifelong blindness on murine neuroanatomy and gene expression. Front Aging Neurosci.

[223] Toldi J, Farkas T, Völgyi B (1994). Neonatal enucleation induces cross-modal changes in the barrel cortex of rat. A behavioural and electrophysiological study. Neurosci Lett.

[224] Bronchti G (2002). Auditory activation of “visual” cortical areas in the blind mole rat (Spalax ehrenbergi). Eur J Neurosci.

[225] Chabot N (2008). Subcortical auditory input to the primary visual cortex in anophthalmic mice. Neurosci Lett.

[226] Karlen SJ, Kahn DM, Krubitzer L (2006). Early blindness results in abnormal corticocortical and thalamocortical connections. Neuroscience.

[227] Charbonneau V, Laramée ME, Boucher V, Bronchti G, Boire D (2012). Cortical and subcortical projections to primary visual cortex in anophthalmic, enucleated and sighted mice. Eur J Neurosci.

[228] Olcese U, Iurilli G, Medini P (2013). Cellular and synaptic architecture of multisensory integration in the mouse neocortex. Neuron.

[229] Sur M, Leamey CA (2001). Development and plasticity of cortical areas and networks. Nat Rev Neurosci.

[230] Lyckman AW (2001). Enhanced plasticity of retinothalamic projections in an ephrin-A2/A5 double mutant. J Neurosci.

[231] Telley L (2019). Temporal patterning of apical progenitors and their daughter neurons in the developing neocortex. Science.

[232] Badia-I-Mompel P (2023). Gene regulatory network inference in the era of single-cell multi-omics. Nat Rev Genet.

[233] Haghverdi L, Ludwig LS (2023). Single-cell multi-omics and lineage tracing to dissect cell fate decision-making. Stem Cell Reports.

[234] Cadwell CR (2017). Multimodal profiling of single-cell morphology, electrophysiology, and gene expression using Patch-seq. Nat Protoc.

[235] Yao Z (2023). A high-resolution transcriptomic and spatial atlas of cell types in the whole mouse brain. Nature.

[236] Dooley JC, van der Heijden ME (2024). More Than a Small Brain: The Importance of Studying Neural Function during Development. J Neurosci.

[237] O’Leary DD, Yates PA, McLaughlin T (1999). Molecular development of sensory maps: representing sights and smells in the brain. Cell.

[238] Korematsu K, Redies C (1997). Restricted expression of cadherin-8 in segmental and functional subdivisions of the embryonic mouse brain. Dev Dyn.

[239] Inoue T, Chisaka O, Matsunami H, Takeichi M (1997). Cadherin-6 expression transiently delineates specific rhombomeres, other neural tube subdivisions, and neural crest subpopulations in mouse embryos. Dev Biol.

[240] Suzuki SC, Inoue T, Kimura Y, Tanaka T, Takeichi M (1997). Neuronal circuits are subdivided by differential expression of type-II classic cadherins in postnatal mouse brains. Mol Cell Neurosci.

[241] Nakagawa Y, O’Leary DD (2001). Combinatorial expression patterns of LIM-homeodomain and other regulatory genes parcellate developing thalamus. J Neurosci.

[242] Jin S (2021). Inference and analysis of cell-cell communication using CellChat. Nat Commun.

[243] Engmann AK (2022). Neuronal subtype-specific growth cone and soma purification from mammalian CNS via fractionation and fluorescent sorting for subcellular analyses and spatial mapping of local transcriptomes and proteomes. Nat Protoc.

[244] Sherman SM, Usrey WM (2024). Transthalamic Pathways for Cortical Function. J Neurosci.

